# Laminin-bound integrin α6β4 promotes non-small cell lung cancer progression *via* the activation of YAP/TAZ signaling pathway

**DOI:** 10.3389/fonc.2022.1015709

**Published:** 2022-10-06

**Authors:** Xiaopeng Zhao, Chuang Liu, Xu He, Miao Wang, Haoran Zhang, Jingge Cheng, Hongyan Wang

**Affiliations:** ^1^ Department of Thoracic Surgery, The Fourth Hospital of Hebei Medical University, Shijiazhuang, China; ^2^ Department of Thoracic Surgery, The Fourth Central Hospital of Baoding City, Baoding, China

**Keywords:** laminin, integrin α6β4, nsclc, YAP/TAZ signaling, tumor progression

## Abstract

Laminin is an extracellular matrix multidomain trimeric glycoprotein, that has a potential role in tumor progression. Here, we studied the effects of non-small cell lung cancer (NSCLC) cells interaction on laminin and explored the underlying mechanism of laminin associated NSCLC progression. Culture of A549 and NCI-1299 cells on 2D collagen gels (containing laminin) significantly promoted the proliferative and tumorigenic characteristics, as well as cell invasion of tumor cells *in vitro*. Consistently, comparing the clinical NSCLC tumor tissues, a poor overall survival was observed in patients with high laminin expression. Mechanistically, the expression of integrin α6β4 was required for the pro-tumor effects of laminin. Meanwhile, we showed that the downstream signaling of integrin α6β4, involved the focal adhesion kinase (FAK)/Yes-Associated Protein (YAP)/TAZ signaling pathway. The activation of FAK/YAP/TAZ signaling pathway induced by laminin was validated in tumor tissues from NSCLC patients. Suppression of integrin α6β4/FAK/YAP/TAZ signaling pathway efficiently suppressed the laminin-induced tumor growth, and strengthened the anticancer effects of chemotherapy, describing a novel target for NSCLC treatment.

## Introduction

Lung cancer is one of the most common carcinomas with a leading cause of cancer-associated death worldwide (1). Non-small cell lung cancer (NSCLC), a heterogeneous class of tumors, accounts for approximately 85% of diagnosed lung cancer cases (2). Patients with NSCLC are usually diagnosed with advance-stage diseases due to the late onset of clinical characteristics. Consequently, NSCLC patients exhibited a very poor prognosis, despite optional management strategies (3). And there is urgent demand to expound the underlying mechanisms of NSCLC development and explore novel therapeutic targets for tumor diagnose or therapy.

Cancer cells’ activity is regulated by diverse factors, including the mutations of oncogenes, tumor microenvironment and extracellular matrix (ECM) (4). Increasing evidence suggests that alterations in ECM are involved in the initiation and growth in a diverse of tumor types (5). The components in ECM, such as collagen (6) and fibronectin (7), are believed to promote the cancer stem cells proliferation, and participate in the process of cancer metastasis. In fact, aberrant expression of collagen and fibronectin has been reported to correlate with the poor prognosis of several cancer types, including hepatocellular and urological carcinomas (8, 9). Among the components in ECM, laminin is a high-molecular weight (400~900 kDa) protein, which serves as a major component of the basal lamina and proteins network foundation for stromal cells and tissues (10). Though abnormal overexpression of laminin was observed in the migrating edge of tumor tissues, the specific correlation between laminin and tumor progression remains poorly understood (11). Several integrins have been demonstrated to recognize laminin substrates, including α6β1 and α6β4 (12). Intriguingly, aberrant activation of the α6β4 or α6β1 is implicated in tumor cells migration and proliferation (13, 14). And the expression of integrin β1 or β4 is correlated with the poor progression of lung cancer (15, 16). Thus, the crosstalk between laminin and integrin associated cell adhesion reflects a high association between laminin and tumor progression.

Integrins and other major ECM components usually cooperate to activate the pro-survival signaling pathways and promote the stem cell activity in cancer cells (17). Several signaling pathways are involved in the integrins induced tumor progression, including phosphatidylinositol 3-kinase (PI3K)/protein kinase B (AKT), focal adhesion kinase (FAK)/Src and yes-associated protein (YAP)/TAZ signaling pathways (18). YAP and TAZ are highly related transcriptional regulators involved in tumor malignancies (19). Increasing studies implicated that the activation of YAP/TAZ signaling pathway is crucial for tumor initiation and development (20). A series cancer associated extrinsic cues are demonstrated to overrule the YAP-inhibiting status of tumor cells, including the aberrant activation of integrin signals (21). Though YAP/TAZ signaling pathway is well documented as having pro-effects on tumor progression, its role in ECM-integrin associated cell adhesion remains poorly understood. And inhibition of YAP/TAZ signaling potentially represents a novel strategy for cancer therapy.

The aim of our study was to validate the role of laminin in tumor progression and elucidate the underlying mechanism of NSCLC development. Herewith, our study demonstrated that laminin could promote the pro-survival signals activation through an integrin α6β4 dependent manner, which enhanced NSCLC cells proliferation and invasion. We further unveiled a molecular mechanism in which laminin mediated the activation of FAK/YAP/YAZ signaling pathway to regulate NSCLC development. Blockade of integrin/FAK/YAP/YAZ signals efficiently impaired NSCLC progression, which described a novel sight for NSCLC treatment.

## Materials and methods

### Cell line culture and reagents

Human NSCLC cell line A549 and NCI-H1299 (abbreviated as H1299 in figures) were purchased from the American Type Culture Collection (ATCC) and maintained with RMPI 1640 complete culture medium (Gibico, MA, USA) supplemented with 10% fetal bovine serum (FBS, Gibco, MA, USA). Chemotherapeutic cis-platinum (Cis), gemcitabine (GEM), laminin, type I collagen and type I collagenase were purchased from Sigma (CA, USA). YAP inhibitor cytochalasin D and FAK inhibitor Y15 were purchased from MCM (NJ, USA).

### 2D collagen culture

The 2D collagen culture was performed as previously described (22). Briefly, type I collagen was diluted to 2.5 mg/ml with PBS. Subsequently, 25 μl 10 × phosphate-buffered saline (PBS) and 20 μl 1N NaOH solution were added into 250 μl collagen solution and seeded into 24-well plate and mixed thoroughly. After 37°C incubation for 2 hours, the 2D collagen gels were solid and A549 cells were seeded on the 2D collagen gels for 5 days. For 2D collagen (containing laminin) culture, laminin was added into the collagen mixture at a concentration of 5 μg/ml, and tumor cells were seeded on the 2D collagen gels (containing laminin) for 5 days.

### Clinical specimens

Formalin-fixed, paraffin-embedded human NSCLC tumor tissue sections were obtained from the The Fourth Hospital of Hebei Medical University, and divided into high-degree group (H-D, stage I~II) and low-degree group (L-D, stage III~IV) according to the pathological diagnosis. All patients agreed to participate in the study and informed with written consent. The clinical experiments were carried out according to the Declaration of Helsinki. This study was approved by the Ethics Committee of the The Fourth Hospital of Hebei Medical University.

### Cell proliferation and colony formation

A549 and NCI-H1299 cell proliferation was assessed by Cell Counting Kit-8 (CCK-8, Biyuntian, Beijing, China). Briefly, tumor cells were seeded in 96-well plates (2000 cells/well) and cultured with RMPI 1640 complete culture medium supplemented with 10% FBS. Cell proliferation was examined at 0, 24, 48, and 72 hours according to the manufacturer’s protocol and absorbance was quantified at 450 nm by microplate reader (Biorad, NJ, USA). For the colony formation, A549 and NCI-H1299 cells were seeded in 6-well plates at 500 cells/well and cultured in RMPI complete medium in a humidified incubator. After 10 days, colonies were fixed with paraformaldehyde and stained with crystal violet (Biyuntian, Beijing, China). Visible colonies were counted. Each experiment was performed three times independently.

### Transwell analysis

A549 and NCI-H1299 (5 × 10^4^ cells) were seeded in the 8 μm transwell insert (Corning, CA, USA) containing 100 μl culture medium (10% FBS) and the bottom chamber was filled with 500 μl culture medium (20% FBS). After 24 hours, the migrating cells were fixed with paraformaldehyde and stained with crystal violet. Then the migrating cells numbers were counted. Each experiment was performed three times independently.

### Real-time PCR

Total RNA extracted from tumor cells was reversely transcribed into cDNA with a High-Capacity cDNA Reverse Transcription Kit (Thermo Fisher USA). The quantification of mRNA levels was conducted by real-time PCR using SYBR green dye (Thermo, MA, USA). GAPDH was used for normalization. The delta-delta Ct method (2 –∆∆Ct method) was used to calculate the relative fold gene expression of samples. The primers used are listed in **Supplementary Information**. Each experiment was performed three times independently.

### Western blotting

A549 and NCI-H1299 cell lysates were separated on 10% sodium dodecyl sulfate-polyacrylamide gels (SDS-PAGE). The primary antibodies used were against human proteins integrin α6 (1:1000, Abcam, Cambridge, UK), integrin β4 (1:1000, Abcam, Cambridge, UK), YAP (1:1000, Abcam, Cambridge, UK), phosphorylated YAP (1:1000, Abcam, Cambridge, UK), TAZ (1:1000, Abcam, Cambridge, UK), FAK (1:1000, Abcam, Cambridge, UK), phosphorylated FAK (1:1000, Abcam, Cambridge, UK), and β-actin (1:1000, Abcam, Cambridge, UK) was used as control. Each experiment was performed three times independently.

### Cell apoptosis analysis

Cell apoptosis was examined using the FITC-Annexin V and PE-PI apoptosis detection kit (BD, NJ, USA). Briefly, pre-treated tumor cells were collected and stained with FITC-Annexin V and PE-PI staining solution for 15 min at room temperature. Cell apoptosis was then detected by flow cytometry on a C6 flow cytometer (BD, NJ, USA). Each experiment was performed three times independently.

### Immunohistochemical staining

The sections of NSCLC tissues were dewaxed, rehydrated, quenched of endogenous peroxidase, blocked by 5% BSA, and incubated with the primary antibody laminin (1:200; Abcam, Cambridge, UK) or phosphorylated FAK (1:200, Abcam, Cambridge, UK) at 4°C overnight, followed by secondary antibody (Thermo, MA, USA) incubation and counter-staining with hematoxylin. The intensity of protein expression was calculated by IPP pro software.

### Transfection and plasmids

The lentiviral vector plv-EF1a-ITGB4-IRES-puro and plv-EF1a-ITGA6-IRES-puro were constructed using Plv-EF1a-IRES-puro (#85132; Addgene) vector. The EcoRI and BamHI restriction sites were designed for plasmids construction. The target sequences were cloned by Ruibo (Beijing, China). The lentiviral vectors were co-transfected into HEK-293T cells with psPAX2 and pMD2.G at a ratio of 10:5:2 for lentiviral packaging. The culture medium of HEK-293T was used for transfection of A549 and NCI-H1299 cells. The protein knockdown was examined by western blotting.

### Animal protocols

Female 6~8 weeks old NOD-SCID mice were purchased from Huafukang (Beijing, China) and raised in SPF room. The animal protocol of this study was approved by the Institutional Animal Care and Use Committee of The Fourth Hospital of Hebei Medical University. The animal studies were conducted in accordance with the Public Health Service Policy and complied with the WHO guidelines for the humane use and care of animals. In our study, 10^6^ A549 cells were injected into NOD-SCID subcutaneously (n=6 in each group). When the tumor size reach 500 mm^3^, mice were treated with PBS, Cis (10mg/kg), GEM (5 mg/kg), Y15 (5 mg/kg) or combining therapy every two days. The treatment lasted for 2 weeks. Tumor volume was measured, and the survival of mice was recorded every day. The calculation formula of tumor volume is: tumor volume = length × width ^2^/2.

### Statistical analysis

502 NSCLC patients’ information in TCGA database was downloaded from http://ualcan.path.uab.edu/index.html. Results in our study were presented as the mean ± SEM and statistical significance was analyzed using GraphPad 5.0 software. Statistical significance was analyzed by Student’s t-test for two groups or by one-way ANOVA for more than two groups, followed by Tukey’s *post hoc* test. The overall survival was evaluated by Kaplan–Meier survival analysis in R 2.0 or GraphPad 5.0 software. *p < 0.05; **p < 0.01; ns, no significantdifference. Each experiment was performed three times independently.

## Results

### Laminin expression correlated with NSCLC progression

**Figure 1:**
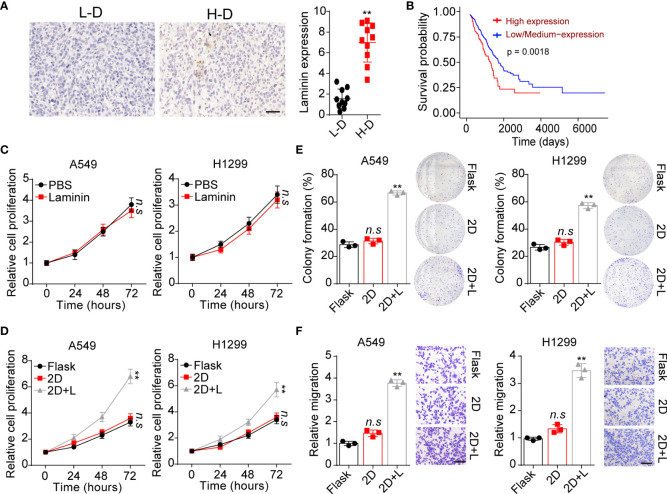
Laminin expression correlated with NSCLC progression. **(A)**, immunohistochemical staining of laminin in high degree (H-D) and low degree (L-D) tumor tissues from NSCLC patients. The scale bar is 50 μm. The intensity of laminin expression was calculated in each group (n=10). **(B)**, overall survival of NSCLC patients divided into laminin expression (n=126) and low/medium laminin expression (n=376) groups using TCGA database analysis. **(C)**, A549 and NCI-H1299 cells were treated with laminin (5 μg/ml) or PBS for 5 days. Then the proliferation of A549 and NCI-H1299 cells was evaluated using CCK-8 analysis. **(D)**, A549 and NCI-H1299 cells were cultured on flask, 2D collagen gels or 2D collagen gels containing laminin for 5 days. Then the proliferation of A549 and NCI-H1299 cells were evaluated using CCK-8 analysis. **(E)**, the colony formation analysis of A549 and NCI-H1299 cells in **(D)**. **(F)**, the relative migration cell numbers of A549 and NCI-H1299 cells in **(D)** using transwell analysis. The scale bar is 50 μm. **p < 0.01; n.s, no significant difference.

It has well illustrated that extracellular laminin serves as cell adhesion associated matrix to regulate stromal cells’ behavior. However, the potential role of laminin in NSCLC development remained poorly understood. Here, tumor tissues isolated from NSCLC patients were collected and divided into high degree (H-D, stage III~IV) and low degree (L-D, stage I~II) according to the pathological diagnosis. We analyzed the tissues expression of laminin by immunohistochemical staining in H-D and L-D groups. As shown in **Figure 1A**, elevated expression of laminin was found in tumor tissues from H-D NSCLC patients compared with L-D degree group. Furthermore, the overall survival of NSCLC patients was evaluated by TCGA database, confirming a decreased survival time of patients with high laminin expression (**Figure 1B**), indicating a positive correlation between laminin expression and NSCLC progression. To further explore the role of laminin in NSCLC progression, human NSCLC cell lines A549 and NCI-H1299 cells were used as *in vitro* model systems, which were cultured with RPMI-1640 culture medium containing laminin for 5 days. Subsequently, A549 and NCI-H1299 cells were collected and the proliferation was examined.

Unfortunately, no obvious difference was observed in proliferation of A549 and NCI-H1299 cells treated with laminin or not (**Figure 1C**). Increasing evidence has suggested that laminin is recognized by integrins, that bind exclusively to this ECM substrate to transduce biomechanical force signals (23). Based on this knowledge, we supposed that solid ECM substrate could confer biomechanical force to laminin, which participate in the tumor progression regulation through transducing biomechanical force associated signals to tumor cells. Thus, we further cultured tumor cells with 2D collagen gels (containing laminin). Intriguingly, 2D collagen (containing laminin) culture significantly potentiated the proliferative characteristics of A549 and NCI-H1299 cells, whereas sole 2D collagen culture exhibited no influence on NSCLC cells comparing with flask culture (**Figure 1D**). Next, we wondered whether the tumor cells cultured with laminin can obtain strengthened tumorigenic/invasive potential, first, we assessed the spheroid formation capability of tumor cells cultured in different methods. As shown in **Figure 1E**, A549 and NCI-H1299 cultured on 2D collagen gels (containing laminin) obtained enhanced capability to form colonies comparing with sole 2D collagen or flask culture. Meanwhile, we seeded those NSCLC cell lines into Transwell inserts to assess the invasive capability. Consistently, increasing migrating tumor cells were observed in 2D collagen gels (containing laminin) cultured group comparing with control groups (**Figure 1F**). Collectively, those results provided evidence to suggested that laminin could promote tumor cells proliferative/invasive potential to induce NSCLC progression.

### Laminin induced NSCLC development through integrin α6β4 receptor

**Figure 2:**
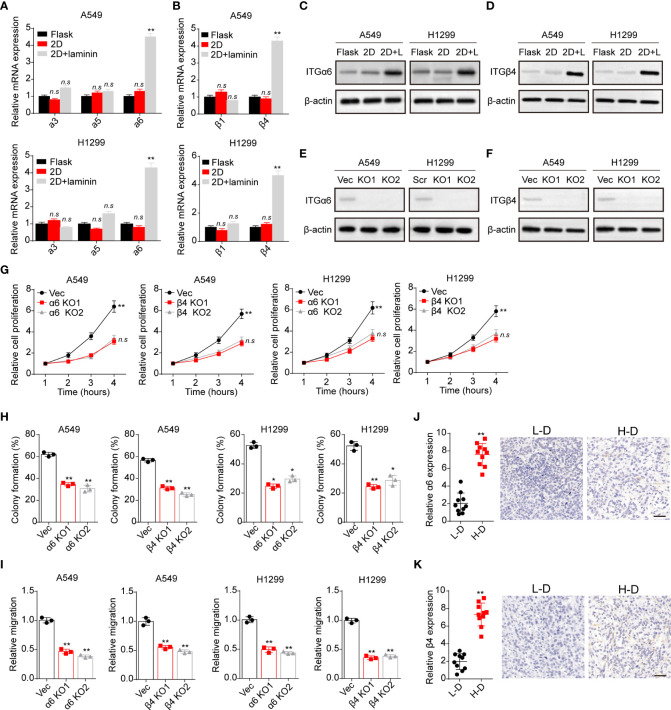
Laminin induced NSCLC development in an integrin α6β4-dependent manner. **(A)**, A549 and NCI-H1299 cells were cultured on flask, 2D collagen gels or 2D collagen gels containing laminin for 5 days. Then the expression of integrin α3, α5 and α6 in A549 and NCI-H1299 cells was detected using real-time PCR. **(B)**, the expression of integrin β1 and β4 in A549 and NCI-H1299 cells in **(A)** was examined. **(C)**, western blotting of integrin α6 in A549 and NCI-H1299 cells in **(A)** was assessed. **(D)**, western blotting of integrin β4 in A549 and NCI-H1299 cells in **(A)** was evaluated. **(E)**, integrin α6 knockdown in A549 and NCI-H1299 cells was evaluated with western blotting. **(F)**, integrin β4 knockdown in A549 and NCI-H1299 cells was evaluated with western blotting. **(G)**, integrin α6 and β4 were knockdown separately in A549 and NCI-H1299 cells. Then cells were cultured on 2D collagen gels containing laminin for 5 days. Next, A549 and NCI-H1299 cells were collected and the cell proliferation in 72 hours was examined by CCK-8 analysis. **(H)**, the colony formation analysis of A549 and NCI-H1299 cells in **(G)**. **(I)** the relative migration cell numbers of A549 and NCI-H1299 cells in **(G)** using transwell analysis. **(J)**, immunohistochemical staining of integrin α6 in high degree (H-D) and low degree (L-D) tumor tissues from NSCLC patients. The scale bar is 50 μm. **(K)** immunohistochemical staining of integrin β4 in high degree (H-D) and low degree (L-D) tumor tissues from NSCLC patients. The scale bar is 50 μm. *p < 0.05; **p < 0.01; n.s, no significant difference.

Compelling finding suggested that laminin is mainly recognized by integrins that contain α3, α5, α6, β1 and β4 subunits, which bind to this ECM substrate and participating in the tumor development regulation (23). Hence, we detected the expression of integrin α3, α5, α6, β1 and β4 in A549 and NCI-H1299 cells cultured in different methods. Intriguingly, tumor cells cultured on 2D collagen gels (containing laminin) exhibited elevated integrin α6 and β4 expression as detected as real-time PCR analysis (**Figures 2A, B**). Consistently, a remarkably high expression of integrin α6 and β4 in 2D collagen gels (containing laminin) cultured NSCLC cells were determined by western blotting (**Figures 2C, D**), indicating that integrin α6β4 might play a role in laminin associated NSCLC progression. To further confirm the crucial role of integrin α6β4, integrin α6 and β4 were silence separately (**Figures 2E, F**), then tumor cells were seeded on 2D collagen gels (containing laminin). Indeed, knockdown of integrin α6 or β4 efficiently suppressed the proliferative characteristics of 2D collagen gels (containing laminin) cultured A549 and NCI-H1299 cells (**Figure 2G**). Meanwhile, 2D collagen gels (containing laminin) cultured A549 and NCI-H1299 cells revealed weakened capability of colony formation (**Figure 2H**) and invasion (**Figure 2I**) when inhibiting integrin α6 and β4, indicating that laminin facilitated NSCLC cells proliferation through integrin α6β4. Subsequently, we further confirmed the expression of integrin α6β4 in tumor tissues from NSCLC patients. Consistently, enhanced expression of integrin α6 (**Figure 2J**) and β4 (**Figure 2K**) was found in H-D tumor tissues from patients. Taken together, these data suggested a critical role of integrin α6β4 in laminin induced NSCLC progression.

### Laminin induced YAP/TAZ activation through integrin α6β4

**Figure 3:**
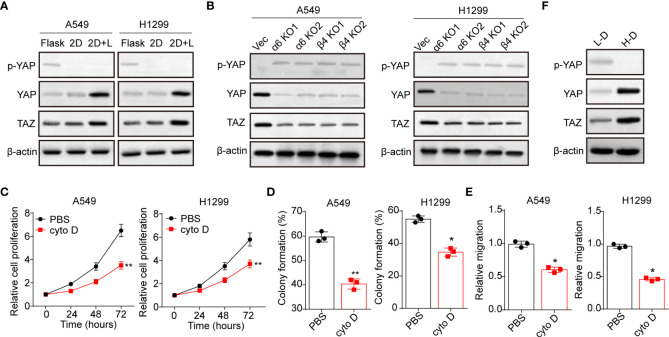
Laminin induced YAP/TAZ activation through integrin α6β4. **(A)**, A549 and NCI-H1299 cells were cultured on flask, 2D collagen gels or 2D collagen gels containing laminin for 5 days. Then the expression of YAP, TAZ and phosphorylated YAP were assessed by western blotting. **(B)**, A549/NCI-H1299, integrin α6 knockdown A549/NCI-H1299 and integrin β4 knockdown A549/NCI-H1299 cells were cultured on 2D collagen gels containing laminin for 5 days. Then the expression of YAP, TAZ and phosphorylated YAP were examined by western blotting. **(C)**, A549 and NCI-H1299 cells were cultured on 2D collagen gels containing laminin for 5 days. Then the cells were cultured with PBS or cytochalasin **(D)** (1 μg/ml) and the cell proliferation was determined. D, the colony formation capability was assessed in tumor cells in **(C)**. **(E)**, the relative migration cells of tumor cells in **(C)** examined by transwell. **(F)**, western blotting of YAP, TAZ and phosphorylated YAP in high degree (H-D) and low degree (L-D) tumor tissues from NSCLC patients. *p < 0.05; **p < 0.01.

Next, we sought to explore the underlying mechanism of laminin induced NSCLC progression. Increasing evidence suggested that activation of YAP/TAZ associated hippo signaling plays a critical role in regulating tumorigenesis, which is tightly correlated with integrins associated signaling pathways activation (24). Thus, we examined whether laminin acts as extracellular cue to mediate the activation of YAP/TAZ signals in NSCLC. We found elevated expression of YAP and TAZ in 2D collagen gels (containing laminin) cultured A549 and NCI-H1299 cells, and reduced cytoplasmic phosphorylated YAP, indicating the activation of YAP/TAZ signals in NSCLC cells (**Figure 3A**). Intriguingly, silence of integrin α6 and β4 suppressed the upregulation of YAP/TAZ signals by laminin (**Figure 3B**), suggesting that laminin induced YAP/TAZ signals activation was integrin α6β4 dependent. To further confirm the role of YAP/TAZ signals in NSCLC progression, we applied cytochalasin D, an inhibitor of actin polymerization that could promote YAP phosphorylation and abort the nucleus entry of YAP, to treat A549 and NCI-H1299 cells. Here, cytochalasin D strongly suppressed the proliferation of 2D collagen gels (containing laminin) cultured A549 and NCI-H1299 cells (**Figure 3C**). Meanwhile, weakened capability of colony formation (**Figure 3D**) and invasion (**Figure 3E**) was found in cytochalasin D treated NSCLC cells, suggesting that laminin promoted NSLCL development in a YAP/TAZ dependent manner. In consistent, western blotting analysis of tumor tissues also revealed activation of YAP/TAZ signals in H-D NSCLC patients comparing with L-D group (**Figure 3F**). These results suggested that laminin facilitated the YAP/TAZ signals activation to promote NSCLC progression.

### Laminin induced YAP/TAZ activation was FAK dependent

**Figure 4:**
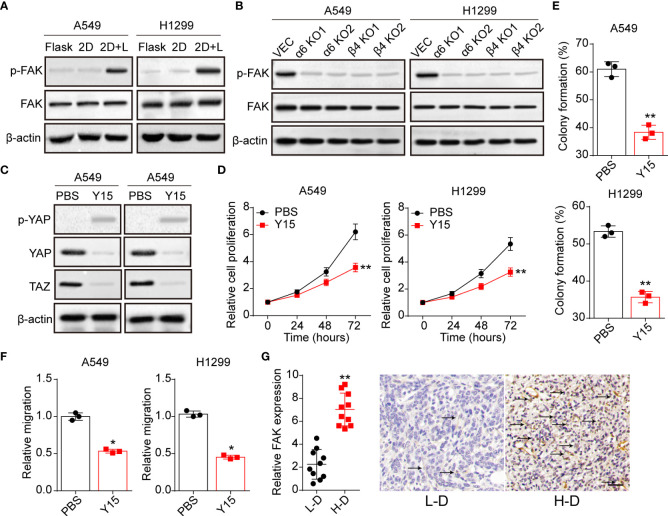
Laminin-induced YAP/TAZ activation is FAK-dependent. **(A)**, A549 and NCI-H1299 cells were cultured on flask, 2D collagen gels or 2D collagen gels containing laminin for 5 days. Then the expression of FAK and phosphorylated FAK were determined by western blotting. **(B)**, A549/NCI-H1299, integrin α6 knockdown A549/NCI-H1299 and integrin β4 knockdown A549/NCI-H1299 cells were cultured on 2D collagen gels containing laminin for 5 days. Then the expression of FAK and phosphorylated FAK were examined by western blotting. **(C)**, A549 and NCI-H1299 cells were cultured on 2D collagen gels containing laminin for 5 days, and PBS or Y15 (1 μM) was added into the culture medium. Then cells were collected and the expression of YAP, TAZ and phosphorylated YAP were determined by western blotting. **(D)**, tumor cells in **(C)** were collected and the proliferation was examined by CCK-8 analysis. E, tumor cells in **(C)** were collected and the colony formation capability was assessed. **(F)**, tumor cells in **(C)** were collected and the relative migrating cell numbers were examined by transwell analysis. **(G)**, immunohistochemical staining of phosphorylated FAK in high degree (H-D) and low degree (L-D) tumor tissues from NSCLC patients. The scale bar is 50 μm. *p < 0.05; **p < 0.01.

Previous studies in epithelial cells suggested that nucleus entry of YAP was promoted by a FAK/Src cascade in response to integrins associated ECM adhesion (25). And the activation of FAK could stabilize YAP levels with reduced S124 and S397 phosphorylation in intestinal stem cells (26). Here, we further examined the expression of FAK in A549 and NCI-H1299 cultured with different methods. Notably, obvious phosphorylated FAK was observed in A549 and NCI-H1299 cultured on 2D collagen gels (containing laminin) (**Figure 4A**), and suppression of integrin α6 or β4 aborted the activation of FAK (**Figure 4B**). Subsequently, we applied Y15, a FAK phosphorylation inhibitor, to treat 2D collagen gels (containing laminin) cultured NSCLC cells, then the expression of YAP/TAZ was examined. Consistent to our hypothesis, Y15 treatment suppressed the activation of YAP/TAZ signals in A549 and NCI-H1299 cultured on 2D collagen gels (containing laminin) (**Figure 4C**), suggesting that YAP/TAZ activation was FAK dependent. Furthermore, we found that Y15 suppressed the pro-tumor effects induced by laminin and YAP/TAZ, as evidenced by the weakened cell proliferation (**Figure 4D**), colony formation (**Figure 4E**) and invasion capability (**Figure 4F**) in 2D collagen gels (containing laminin) cultured A549 and NCI-H1299 cells. Consistently, analysis of immunohistochemistry revealed reduced FAK expression in L-D tumor tissues from NSCLC patients compared with H-D group (**Figure 4G**). Together, those results suggested that laminin mediated YAP/TAZ signals activation through FAK to regulate NSCLC progression.

### Inhibition of integrin/FAK/YAP/TAZ signals suppressed the NSCLC development

**Figure 5:**
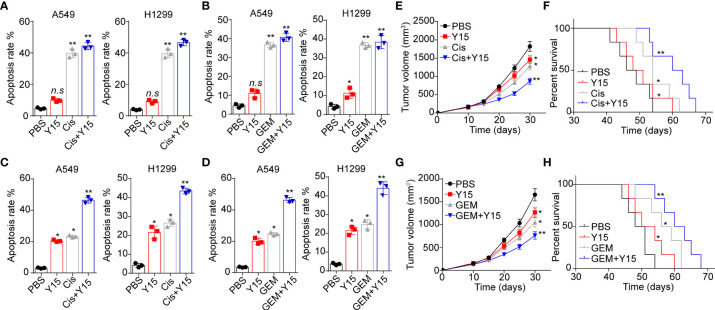
Blockade of integrin/FAK/YAP/TAZ signals suppressed the NSCLC development. **(A)**, A549 and NCI-H1299 cells were cultured on flask and treated with PBS, Y15 (1 μM), Cis (2 μg/ml) and Y15 (1 μM) combing Cis (2 μg/ml). Then the cell apoptosis was determined. **(B)**, A549 and NCI-H1299 cells were cultured on flask and treated with PBS, Y15 (1 μM), GEM (1 μg/ml) and Y15 (1 μM) combing GEM (1 μg/ml). Then the cell apoptosis was examined. **(C)**, A549 and NCI-H1299 cells were cultured on 2D collagen gels containing laminin and treated with PBS, Y15 (1 μM), Cis (2 μg/ml) and Y15 (1 μM) combing Cis (2 μg/ml). Then the cell apoptosis was assessed. **(D)**, A549 and NCI-H1299 cells were cultured on 2D collagen gels containing laminin and treated with PBS, Y15 (1 μM), GEM (1 μg/ml) and Y15 (1 μM) combing GEM (1 μg/ml). Then the cell apoptosis was examined. **(E)**, tumor volume of A549 tumor-bearing mice treated with PBS, Cis, Y15 and Cis combing Y15. **(F)**, overall survival of A549 bearing mice treated with PBS, Cis, Y15 and Cis combing Y15. **(G)**, tumor volume of A549 bearing mice treated with PBS, GEM, Y15 and GEM combing Y15. **(H)**, overall survival of A549 bearing mice treated with PBS, Cis, GEM and GEM combing Y15. *p < 0.05; **p < s0.01; n.s, no significant difference.

Given the role of integrin/FAK/YAP/TAZ in NSCLC development, we conjectured that suppression of integrin/FAK/YAP/TAZ signals by Y15 could restrain tumor growth and improve the outcome of chemotherapy. Here, we applied chemotherapeutic Cis and GEM combining Y15 to treat A549 and NCI-H1299 cells cultured in different methods. Intriguingly, Y15 treatment revealed no significant influence to A549 and NCI-H1299 cells cultured in flask. Similarly, no synergistic effects were found in Y15 combining Cis (**Figure 5A**) or GEM (**Figure 5B**). However, A549 and NCI-H1299 cells cultured on 2D collagen gels (containing laminin) revealed obvious sensitivity to Y15. More importantly, resistance to chemotherapy were observed in NSCLC cells cultured on 2D collagen gels (containing laminin), which might be associated with the drugs resistance induced by YAP/TAZ signaling. Combination of Y15 and chemotherapeutic agents efficiently reverse the chemo-resistance and exhibited strengthened cytotoxicity to tumor cells cultured on 2D collagen gels containing laminin (**Figures 5C, D**), suggesting that blockade of ntegrin/FAK/YAP/TAZ signals by Y15 could suppress the pro-tumor effects induced by laminin. As the extensive presence of laminin in tumor tissues *in vivo*, we further examined the anticancer effects of Y15 in tumor bearing mice *in vivo*. In our study, tumors arising from the subcutaneous implication of A549 cells in NOD-SCID mice was established for anticancer effects analysis *in vivo*. Subcutaneous A549 tumors were established in mice and, then treated with PBS, Cis, Y15 and Cis combing Y15. Both Y-15 and Cis treatment retrained the tumor growth and prolonged the overall survival of A549 bearing mice, and strengthened anticancer effects was found in the combination group (**Figures 5E, F**). The similar results were observed in GEM and Y15 combination (**Figures 5G, H**). collectively, these results suggested that suppression of integrin/FAK/YAP/TAZ signals by Y15 could inhibited the laminin associated tumor progression and improved the anticancer effects of chemotherapy *in vivo*, which described a novel strategy for NSCLC treatment.

## Discussion

Laminin is classically recognized as molecule that contributes to breast cancer cells adhesion and growth (27). The aberrant expression of laminin is found in various tumor types, and correlates with the poor prognosis of esophageal squamous cell carcinoma (28). Notably, enrichment of laminin in Matrigel was demonstrated to promote the tumorigenicity and the drug resistance of 3D cultured tumor cells. However, the role of laminin was rarely reported and the underlying mechanism of laminin associated tumor progression remained controversial. In the present communication, we firstly reported that culture of NSCLC cells on laminin coated 2D collagen gels enhanced the cells proliferation and invasion of NSCLC cells, and provided evidence to suggest the positive correlation between laminin expression and the poor prognosis in NSLCL patients.

To elucidate the role of laminin in tumor progression, we initially added laminin into the culture medium of NSCLC cells directed, however, no pro-tumor effects were observed in laminin treated groups. ECM substrates in solid matrix participated in the tumor progression regulation through the cell adhesion induced by integrins, thereby transducing chemical or biomechanical force signals to tumor cells (29). In fact, solid 3D collagen or fibrin gels have been demonstrated to promote the stem-like features and tumorigenic capability in a diverse of tumor cell types (22, 30), whereas limited effects were observed in soluble collagen or fibrinogen *in vitro*. Thus, we confirmed that solid ECM substrate might confer biomechanical force to laminin, then transduce extracellular force signals to tumor cells. Here, laminin was mixed in the solid 2D collagen gels for cell culture. Consequently, 2D collagen containing laminin retendered cancer cells more able to upregulate integrins and cell proliferation. As investigated in integrins expression, we found that integrin α6β4 was necessary for the NSCLC progression induced by laminin. Consistently, the recognition of laminin by integrins have been reported in several tumor types and the potential role of laminin in tumor progression was emphasized. For instance, elevated expression of laminin mediated motility in the breast cancer cells and correlated with the prognosis of patients (31). meanwhile, IHC staining of laminin in human tumor tissues revealed enriched laminin and integrin α6β4 expression in the interface zone between tumor cells and the extracellular stroma. Here, our study further elaborated the role of laminin in NSCLC development, which was in an integrin α6β4 dependent manner.

We have shown that laminin promoted the proliferative and tumorigenic characteristics of NSCLC cells, and played a role in tumor invasion. The laminin associated mechanism that contributed to proliferative features of cancer cells remained poorly reported. Recent studies have shown that a series of integrins, such as integrin α3, α6, β4 and β6, could facilitate the adhesion to laminin of tumor cells (11). and binding of integrins to ECM substrates are essential for the activation of pro-survival signaling pathways in tumor cells (17). Diverse integrins, especially integrin αvβ3 and α2β1, have been reported to be associated with stem/progenitor characteristics of cancer cells, which further contributing the pro-survival signals activation and tumor growth (32). Despite not have founding the alterations of integrin β3 and β1, it was possible that laminin mediated pro-survival signaling pathways activation through integrin α6β4. In the present study, we further explored the downstream signaling pathways of integrin α6β4 and disclosed a crosstalk between laminin/integrin α6β4 and YAP/TAZ signaling pathway. Specifically, FAK served as the downstream of integrin α6β4, which was upregulated in laminin treated NSCLC cells. As a result, the upregulation of FAK further contributed to the pro-survival YAP/TAZ signaling activation. Importantly, the activation of FAK/TAZ/TAP signals was found in high stage NSCLC tumor tissues. And the poor prognosis of NSCLC patients with high laminin expression might be partially relevant to the activation of YAP signaling. Consistently, in our study, suppression of FAK/YAP/TAZ signaling reduced the pro-tumor effects induced by laminin, and efficiently enhanced the anticancer effects in A549-bearing mice.

In conclusion, our study showed that laminin promoted NSCLC development by modulating the activation of FAK/YAP/TAZ signaling pathway in an integrin α6β4 dependent manner. Inhibition of integrin/FAK/YAP/YAZ signals exhibited improved tumor suppressive effects, providing an innovative approach for NSCLC treatment.

## Data availability statement

The original contributions presented in the study are included in the article/**Supplementary Material**. Further inquiries can be directed to the corresponding author.

## Ethics statement

This study was approved by the Ethics Committee of The Fourth Hospital of Hebei Medical University. The clinical experiments were carried out according to the Declaration of Helsinki. All patients agreed to participate in the study and informed with written consent. The animal study was reviewed and approved by Institutional Animal Care and Use Committee of The Fourth Hospital of Hebei Medical University.

## Author contributions

XZ and CL designed the project and wrote the manuscript. XH and MW performed the experiments. HZ and JC collected the experimental data. HW revised the primary manuscript. All authors contributed to the article and approved the submitted version.

## Acknowledgments

We thank The Fourth Hospital of Hebei Medical University for providing experimental support for this study.

## Conflict of interest

The authors declare that the research was conducted in the absence of any commercial or financial relationships that could be construed as a potential conflict of interest.

## Publisher’s note

All claims expressed in this article are solely those of the authors and do not necessarily represent those of their affiliated organizations, or those of the publisher, the editors and the reviewers. Any product that may be evaluated in this article, or claim that may be made by its manufacturer, is not guaranteed or endorsed by the publisher.
