# Supplementary figures and images for: Laminin-bound integrin α6β4 promotes non-small cell lung cancer progression via the activation of YAP/TAZ signaling pathway
Source: Front Oncol. 2022 Oct 6;12:1015709. doi: 10.3389/fonc.2022.1015709 (PMC9583390; doi:10.3389/fonc.2022.1015709)

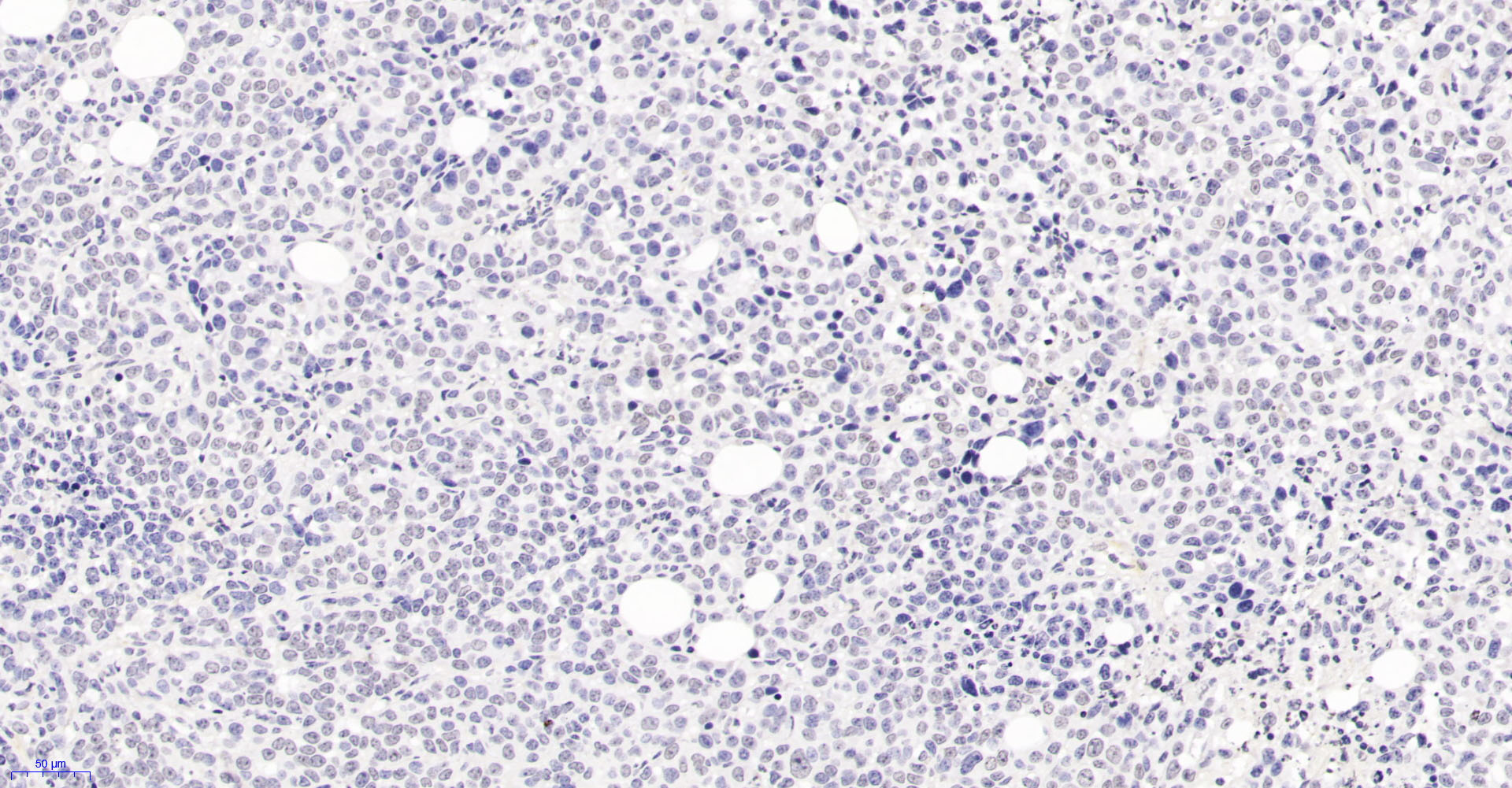

Supplement: Supplementary file 1 [file DataSheet_1.zip › raw data/IHC/FAK HD.jpg]

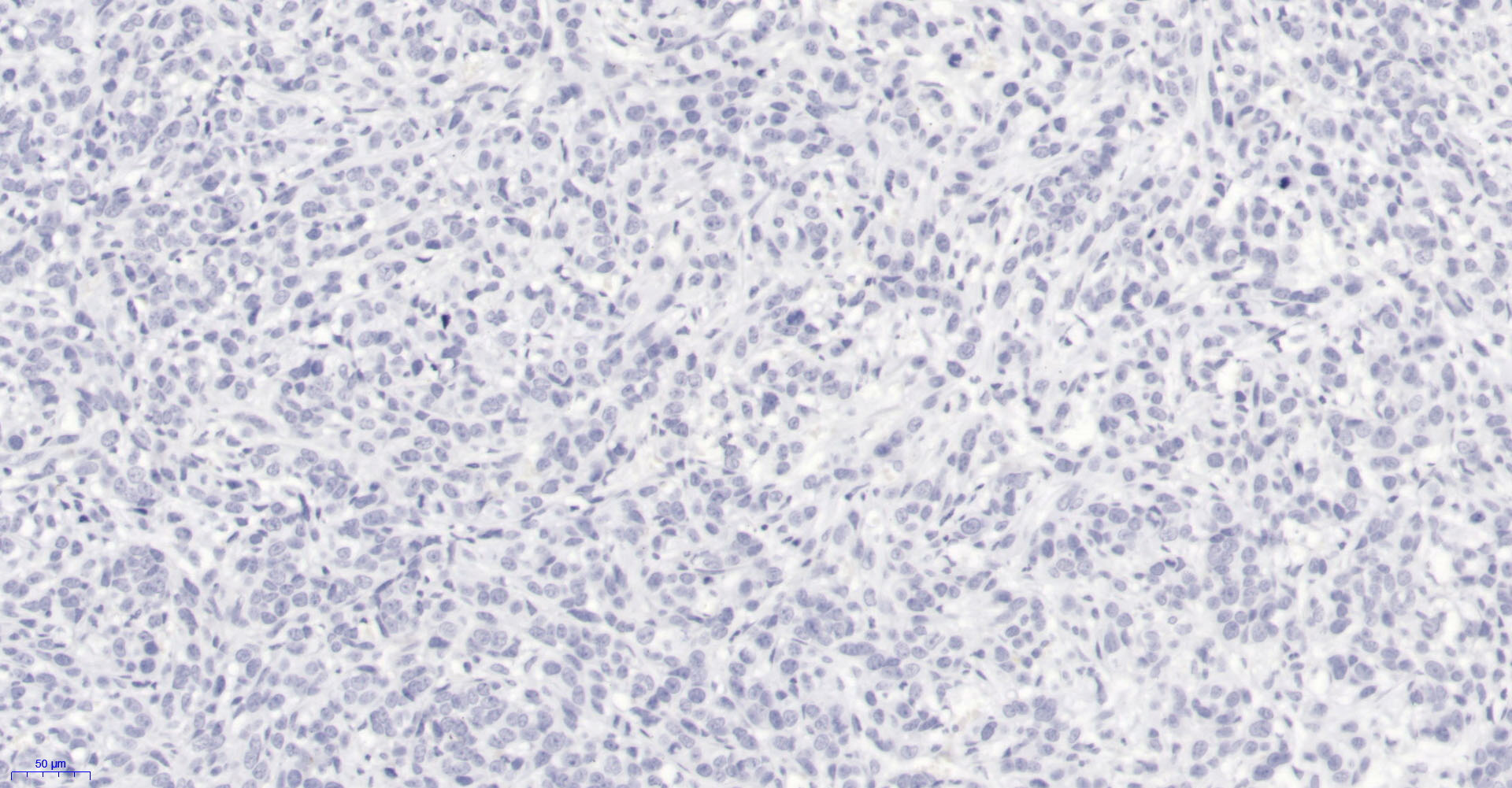

Supplement: Supplementary file 1 [file DataSheet_1.zip › raw data/IHC/FAK LD.jpg]

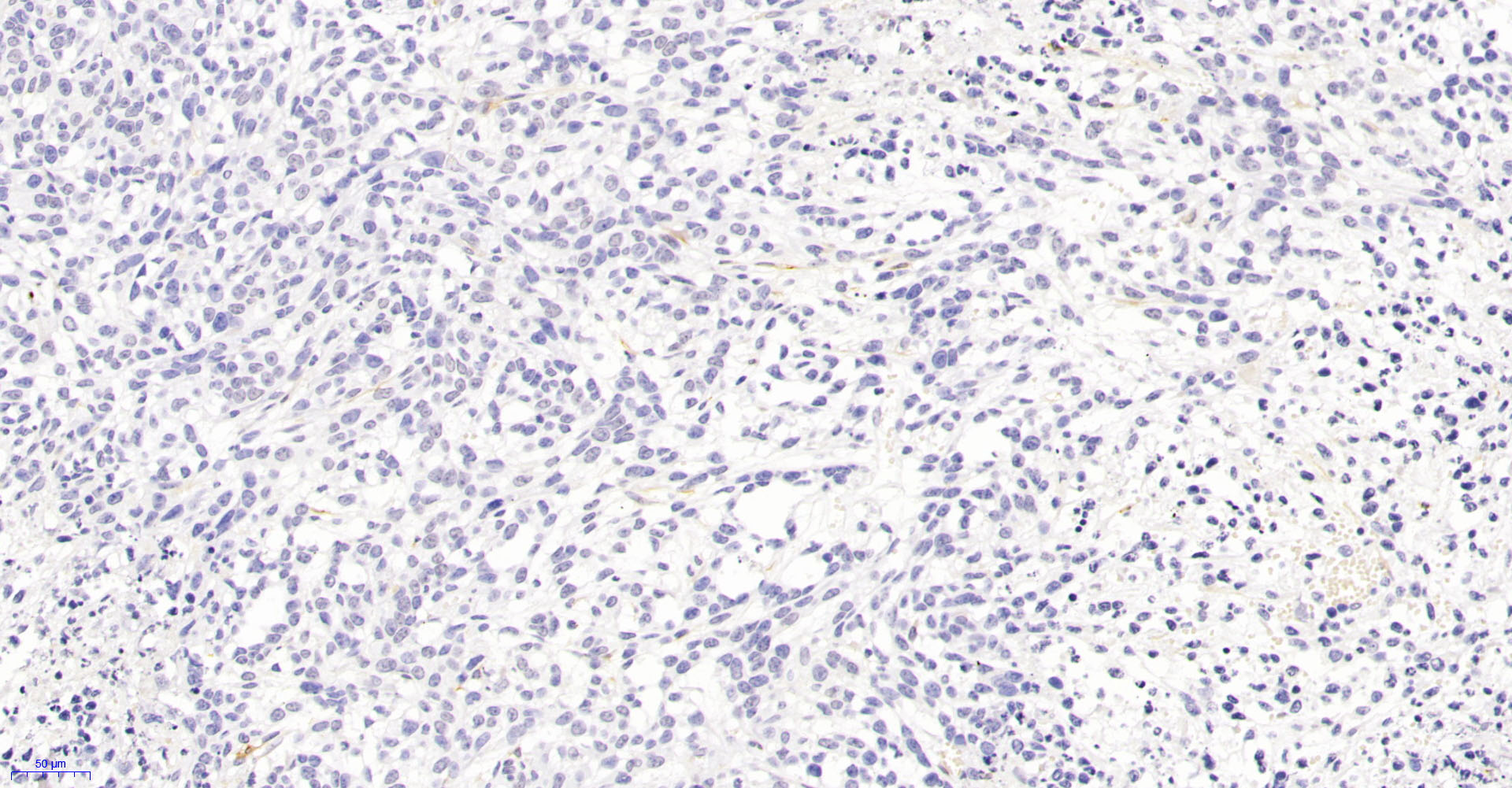

Supplement: Supplementary file 1 [file DataSheet_1.zip › raw data/IHC/ITGA6 HD.jpg]

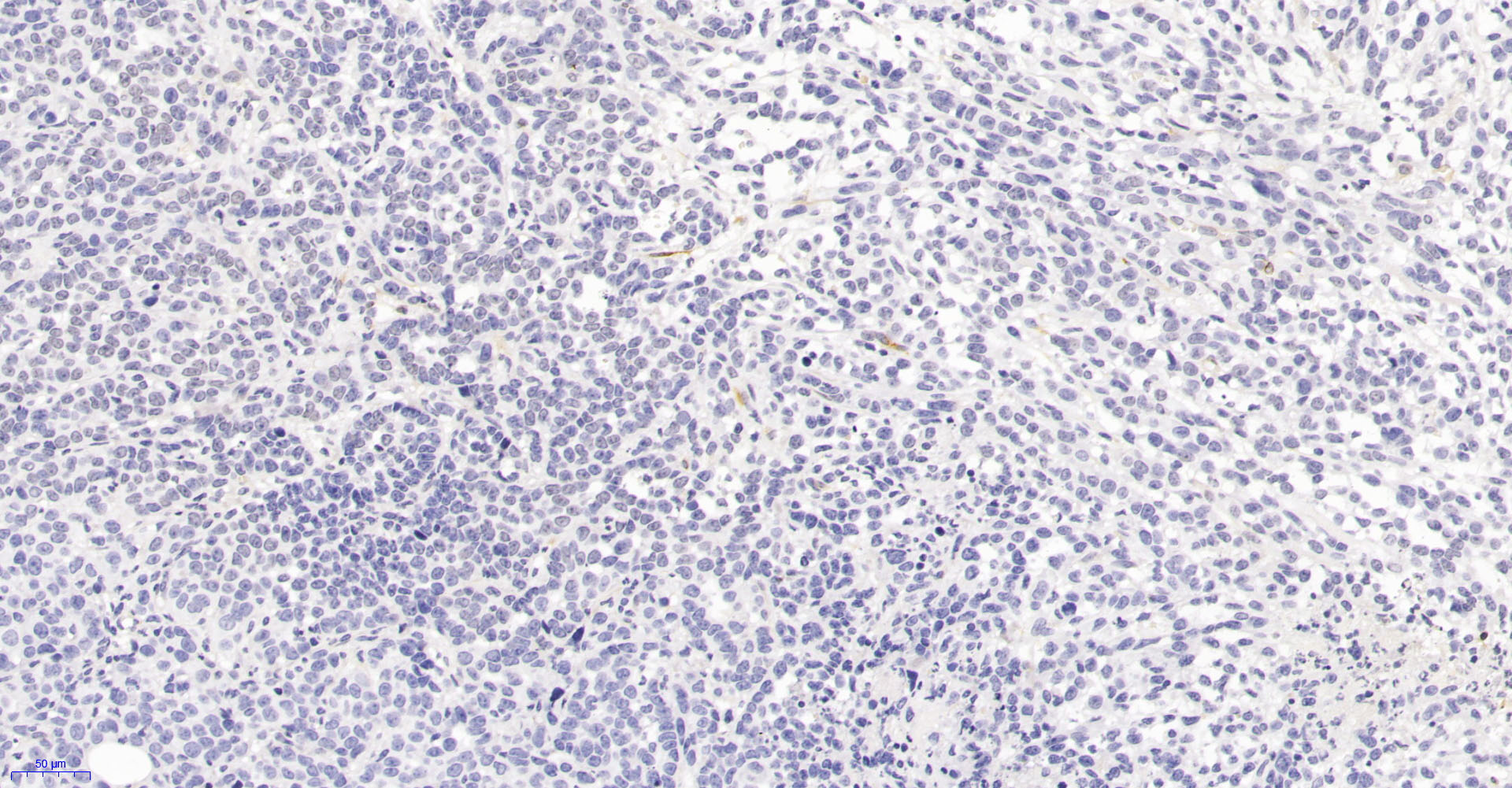

Supplement: Supplementary file 1 [file DataSheet_1.zip › raw data/IHC/ITGA6 LD.jpg]

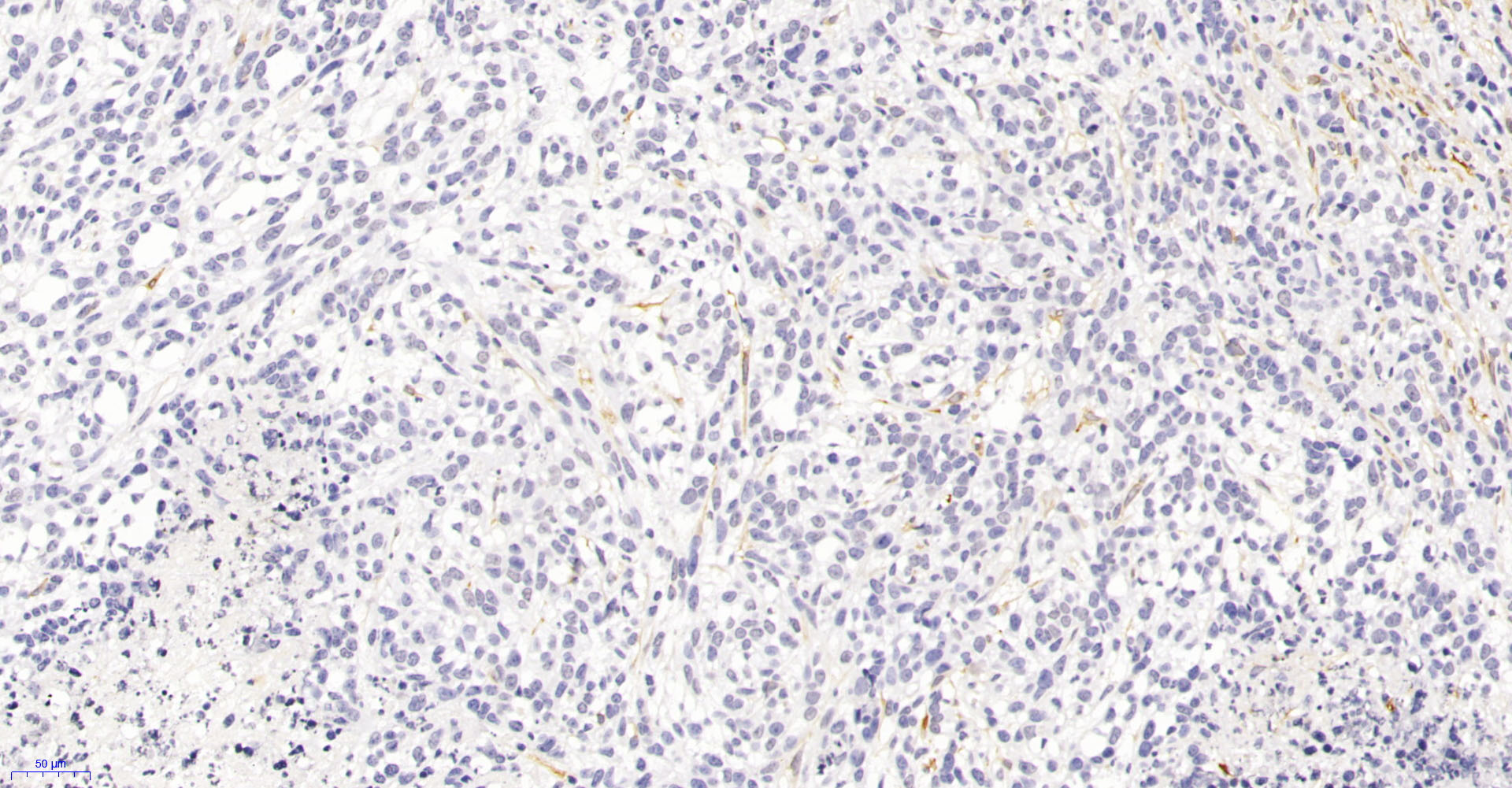

Supplement: Supplementary file 1 [file DataSheet_1.zip › raw data/IHC/ITGB4 HD.jpg]

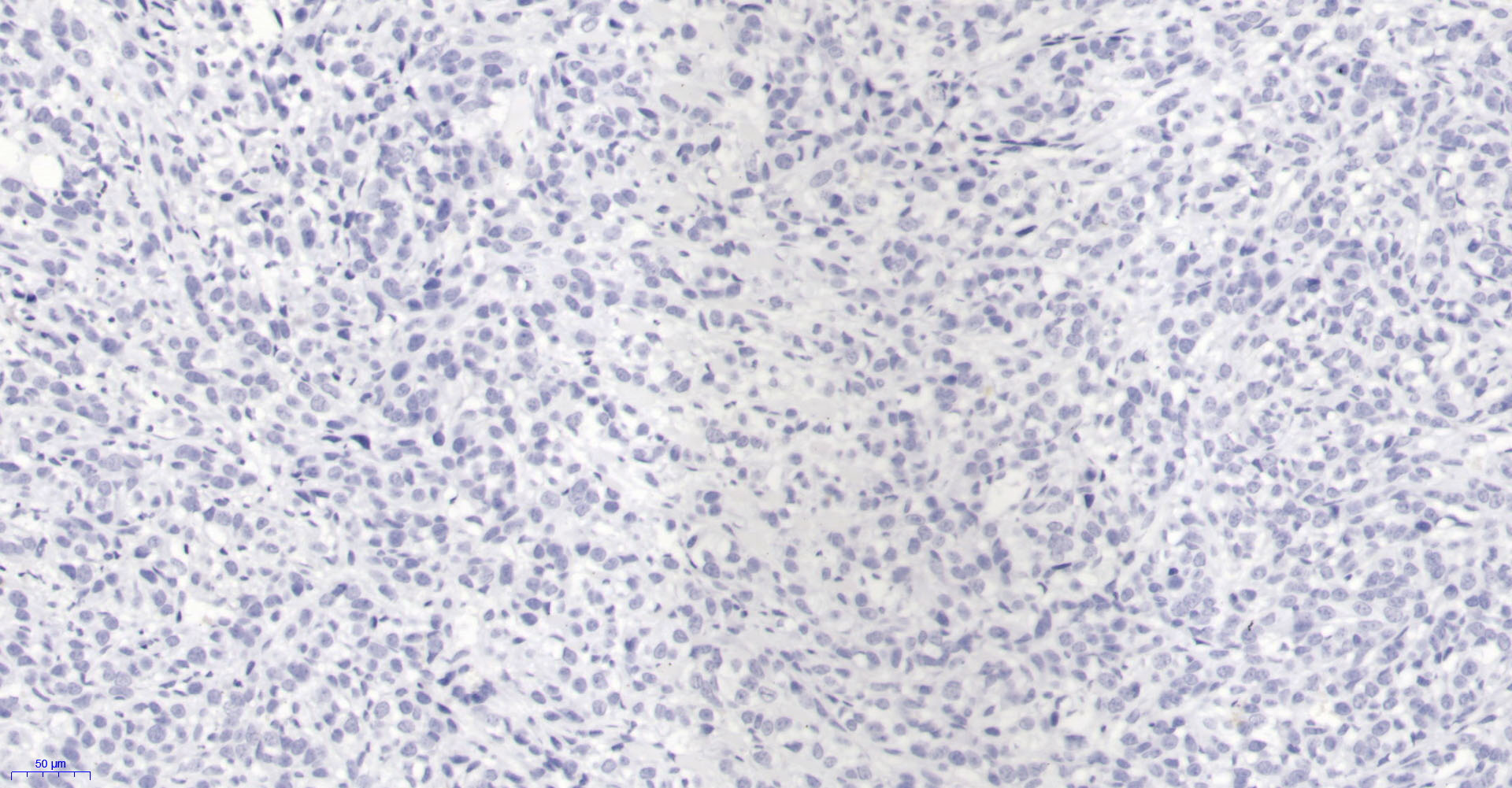

Supplement: Supplementary file 1 [file DataSheet_1.zip › raw data/IHC/ITGB4 LD.jpg]

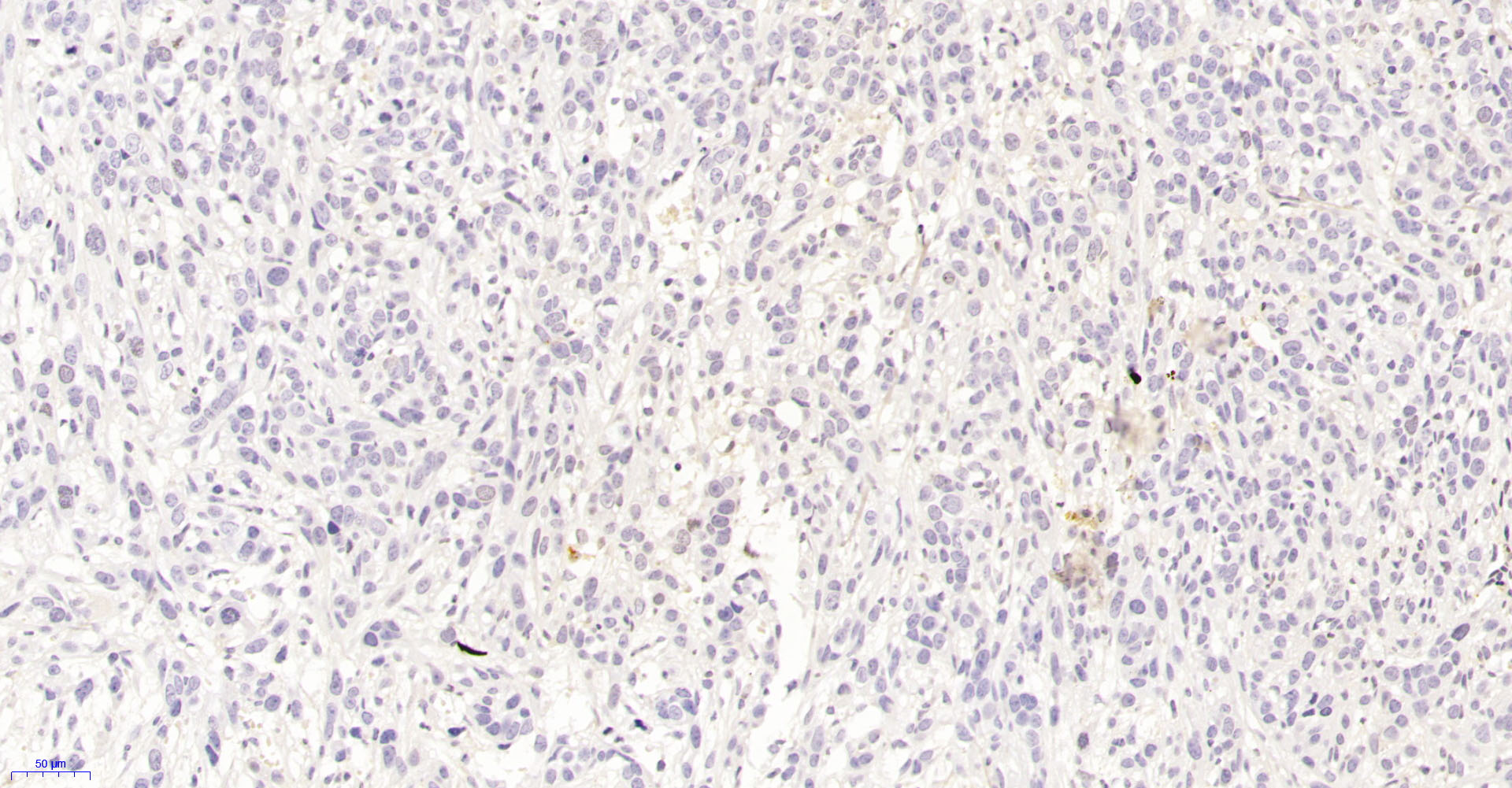

Supplement: Supplementary file 1 [file DataSheet_1.zip › raw data/IHC/laminin HD.jpg]

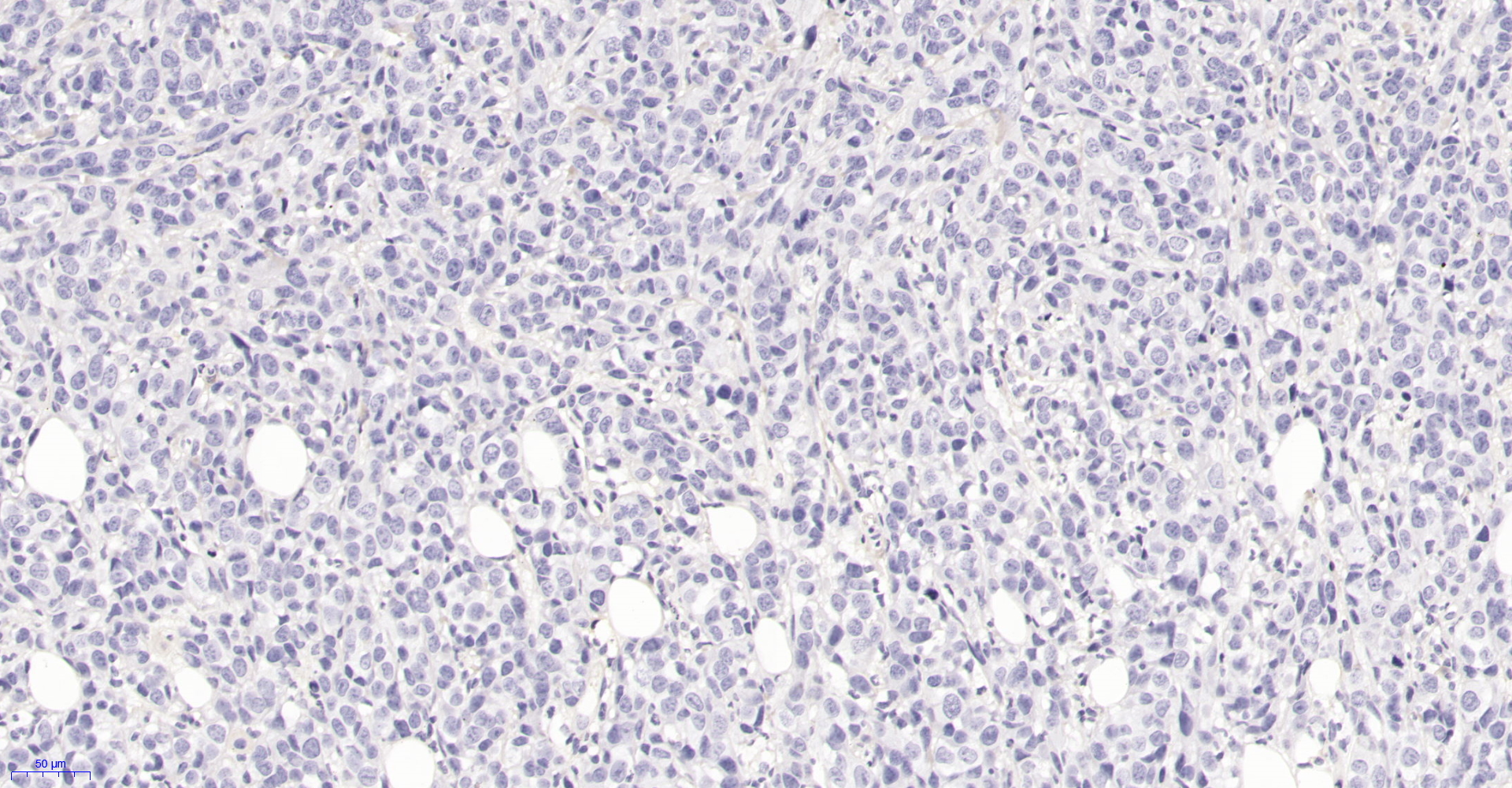

Supplement: Supplementary file 1 [file DataSheet_1.zip › raw data/IHC/laminin LD.jpg]

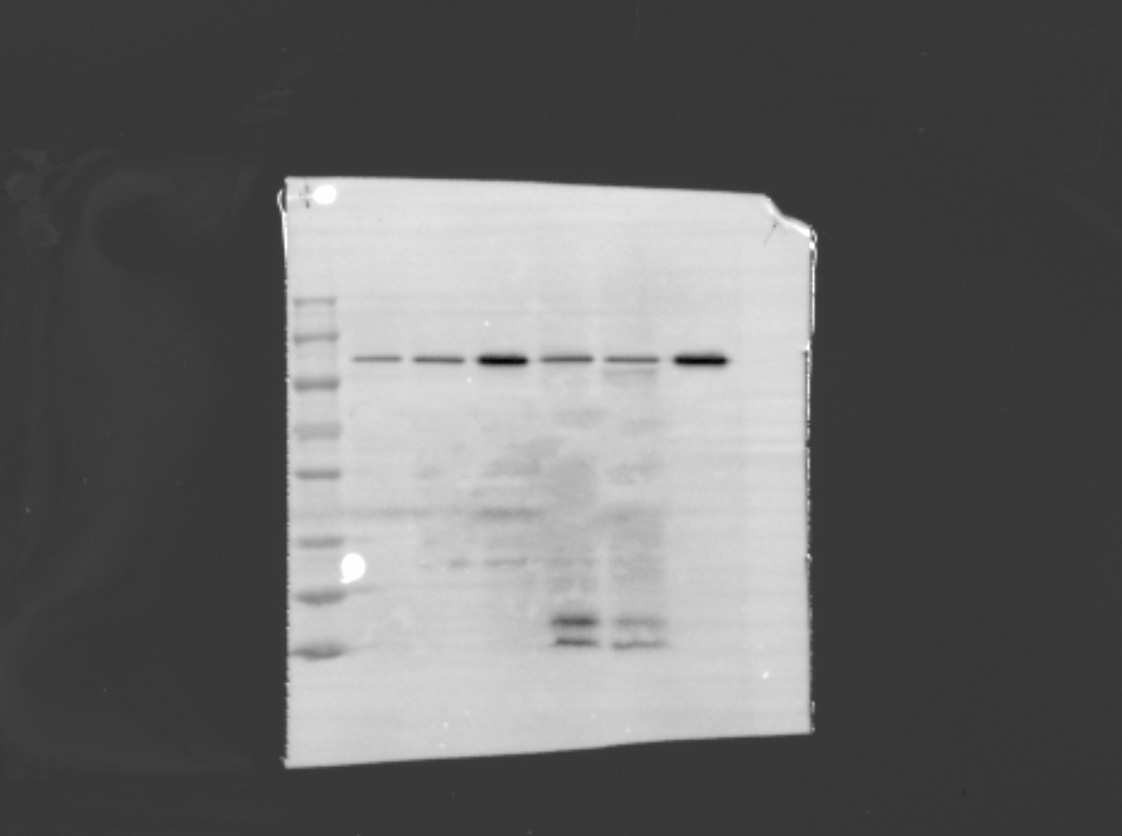

Supplement: Supplementary file 1 [file DataSheet_1.zip › raw data/WB/figure 2/fig. 2C/ITGa6-A549-H1299.jpg]

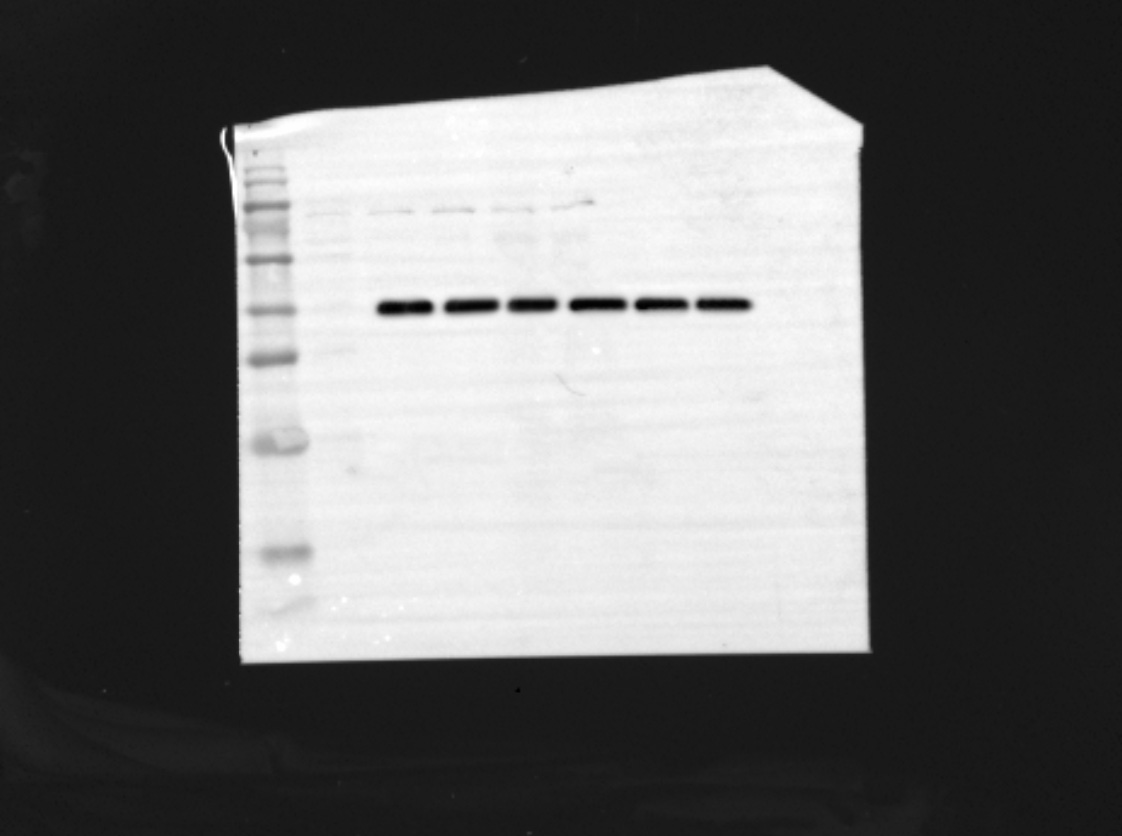

Supplement: Supplementary file 1 [file DataSheet_1.zip › raw data/WB/figure 2/fig. 2C/ITGa6-actin-a549-h1299.jpg]

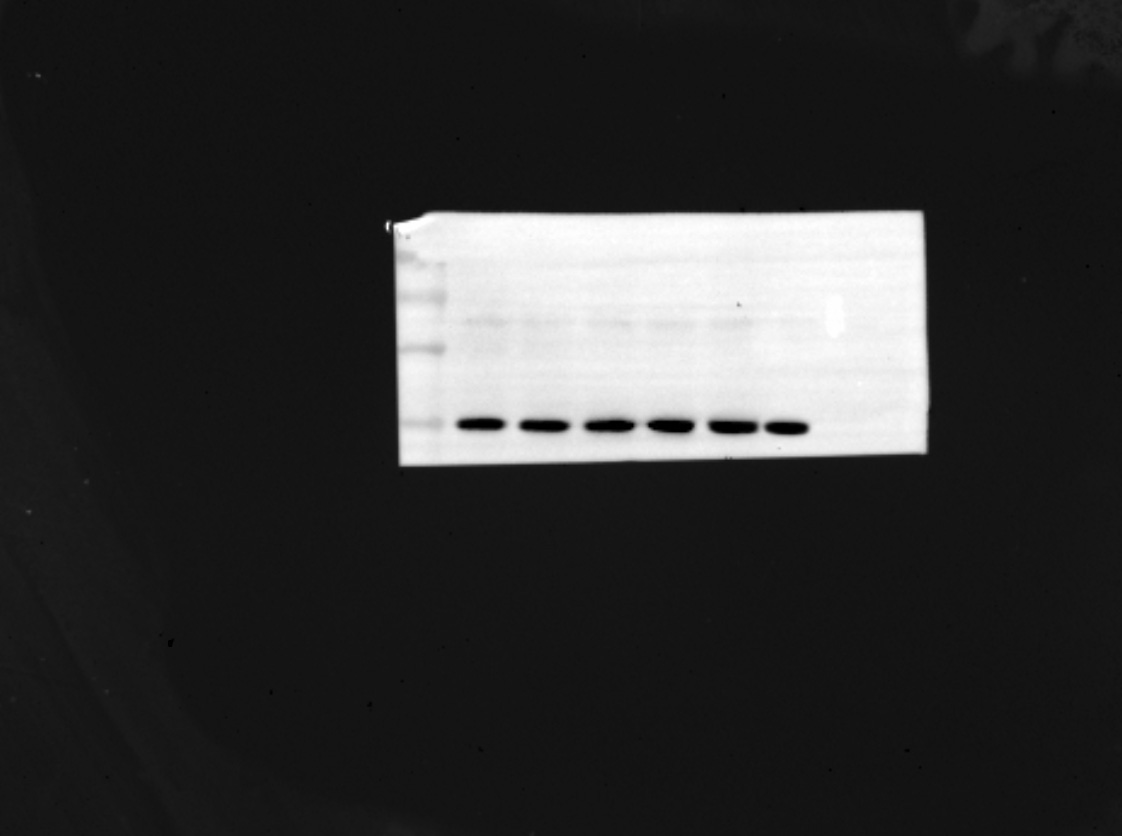

Supplement: Supplementary file 1 [file DataSheet_1.zip › raw data/WB/figure 2/fig. 2E/a6 ko actin.jpg]

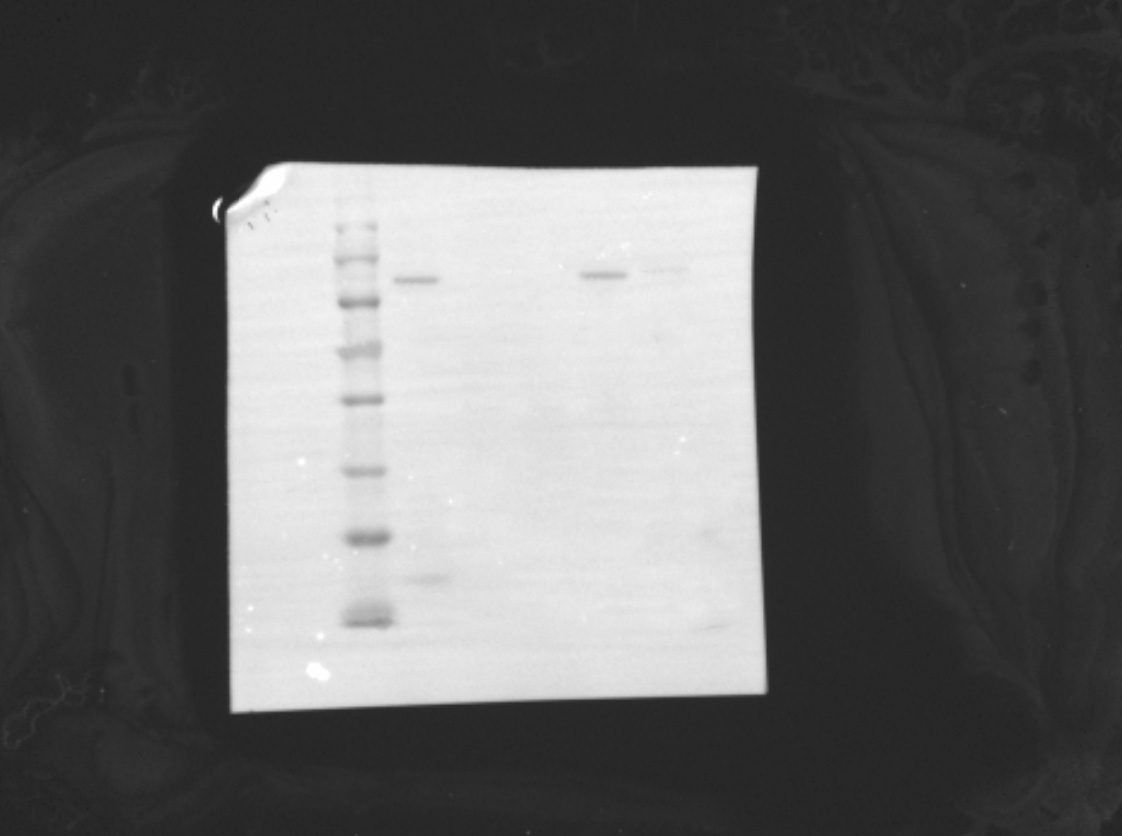

Supplement: Supplementary file 1 [file DataSheet_1.zip › raw data/WB/figure 2/fig. 2E/ITGB a6 ko.jpg]

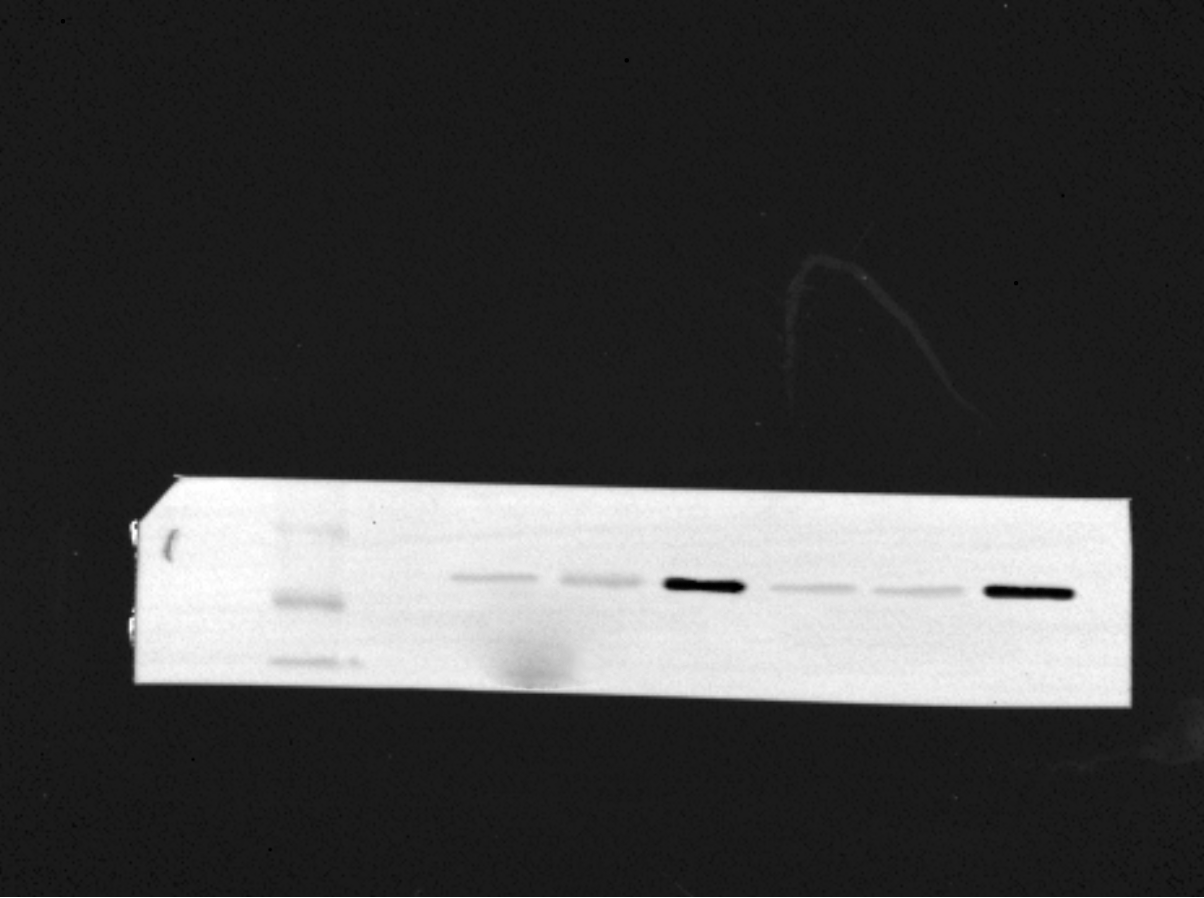

Supplement: Supplementary file 1 [file DataSheet_1.zip › raw data/WB/figure 2/fig.2D/ITGB b4-a549-h1299.tif]

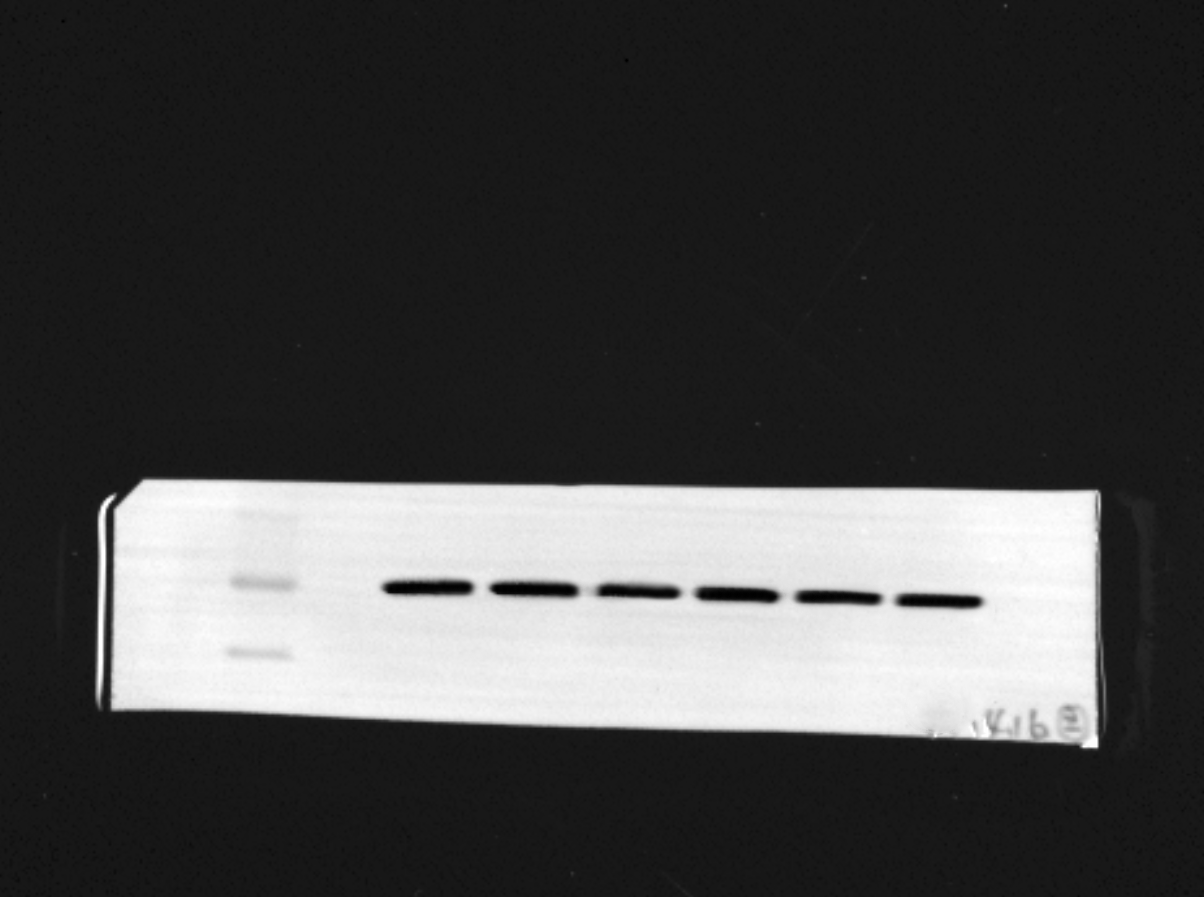

Supplement: Supplementary file 1 [file DataSheet_1.zip › raw data/WB/figure 2/fig.2D/ITGBb4 actin-a549-h1299.tif]

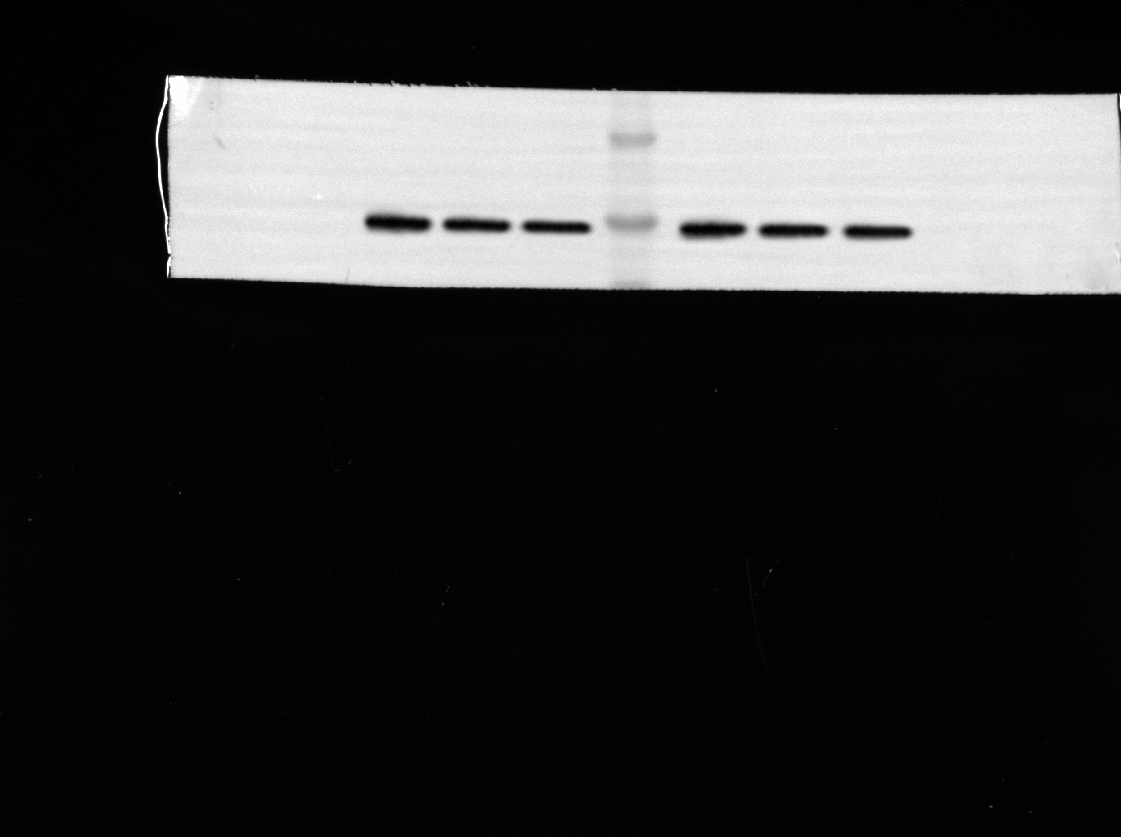

Supplement: Supplementary file 1 [file DataSheet_1.zip › raw data/WB/figure 2/fig.2F/ITGB b4 ko actin.tif]

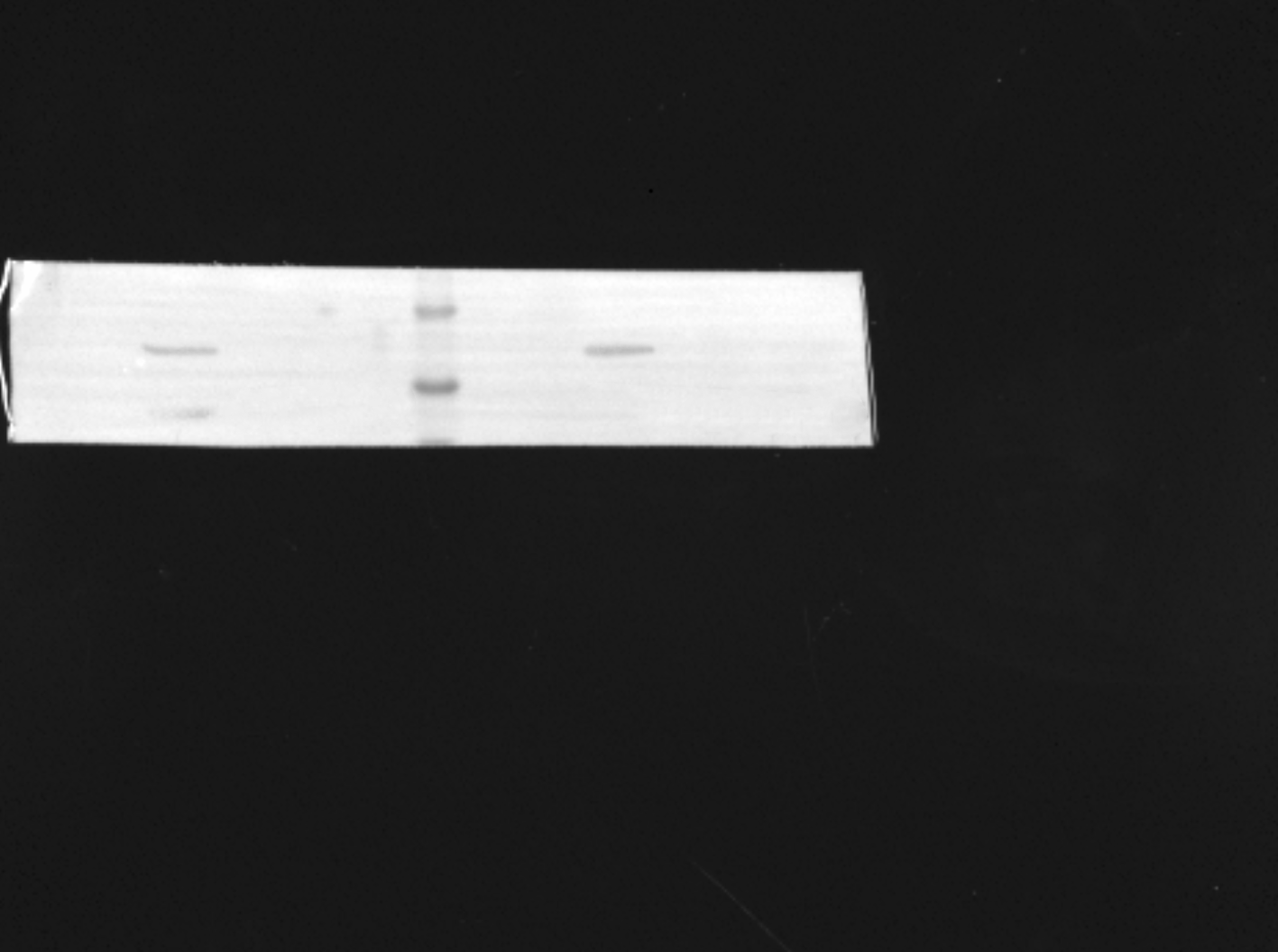

Supplement: Supplementary file 1 [file DataSheet_1.zip › raw data/WB/figure 2/fig.2F/ITGB b4 ko.tif]

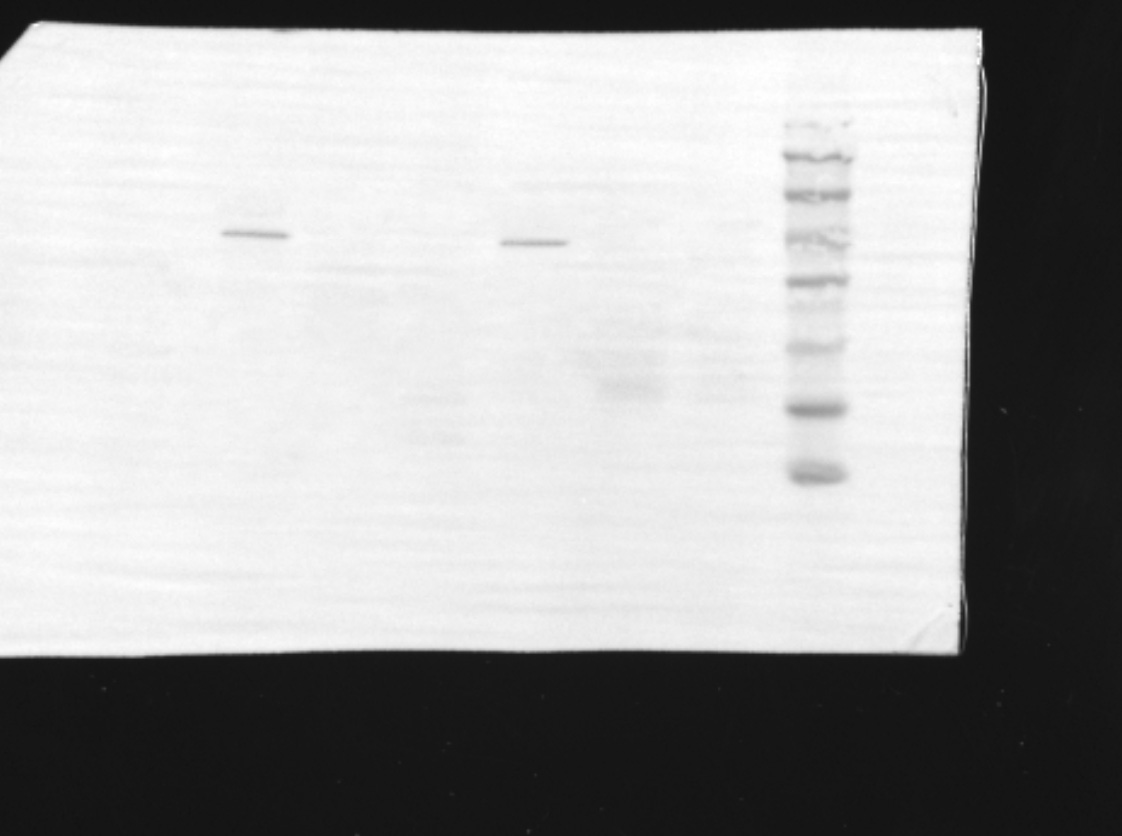

Supplement: Supplementary file 1 [file DataSheet_1.zip › raw data/WB/figure 3/A/p-YAP.jpg]

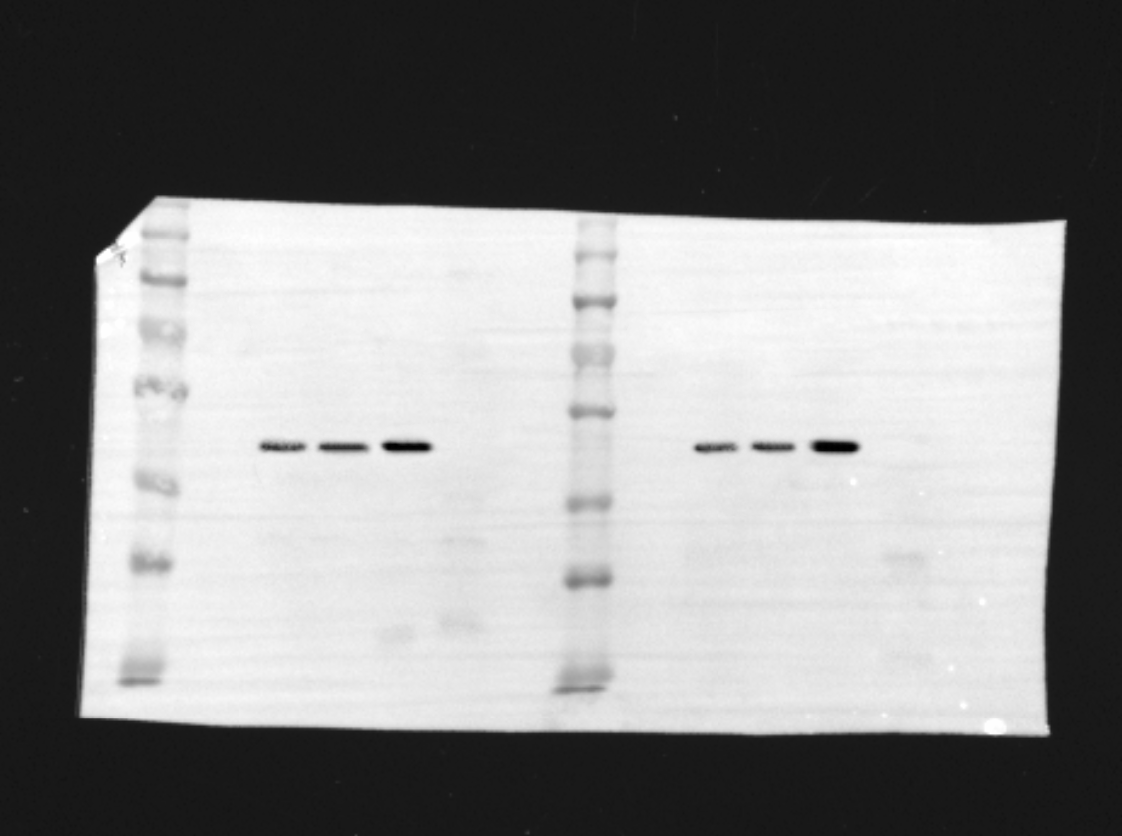

Supplement: Supplementary file 1 [file DataSheet_1.zip › raw data/WB/figure 3/A/TAZ.A549-H1299.tif]

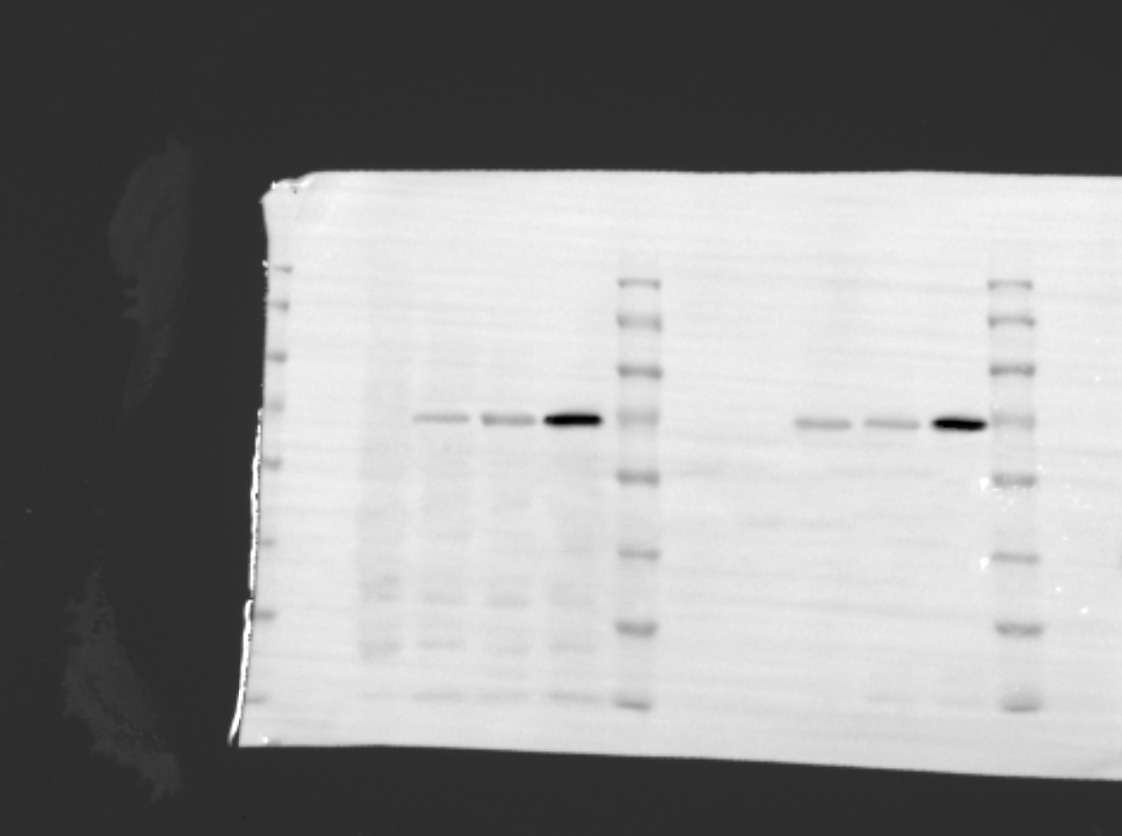

Supplement: Supplementary file 1 [file DataSheet_1.zip › raw data/WB/figure 3/A/YAP-A549-H1299.jpg]

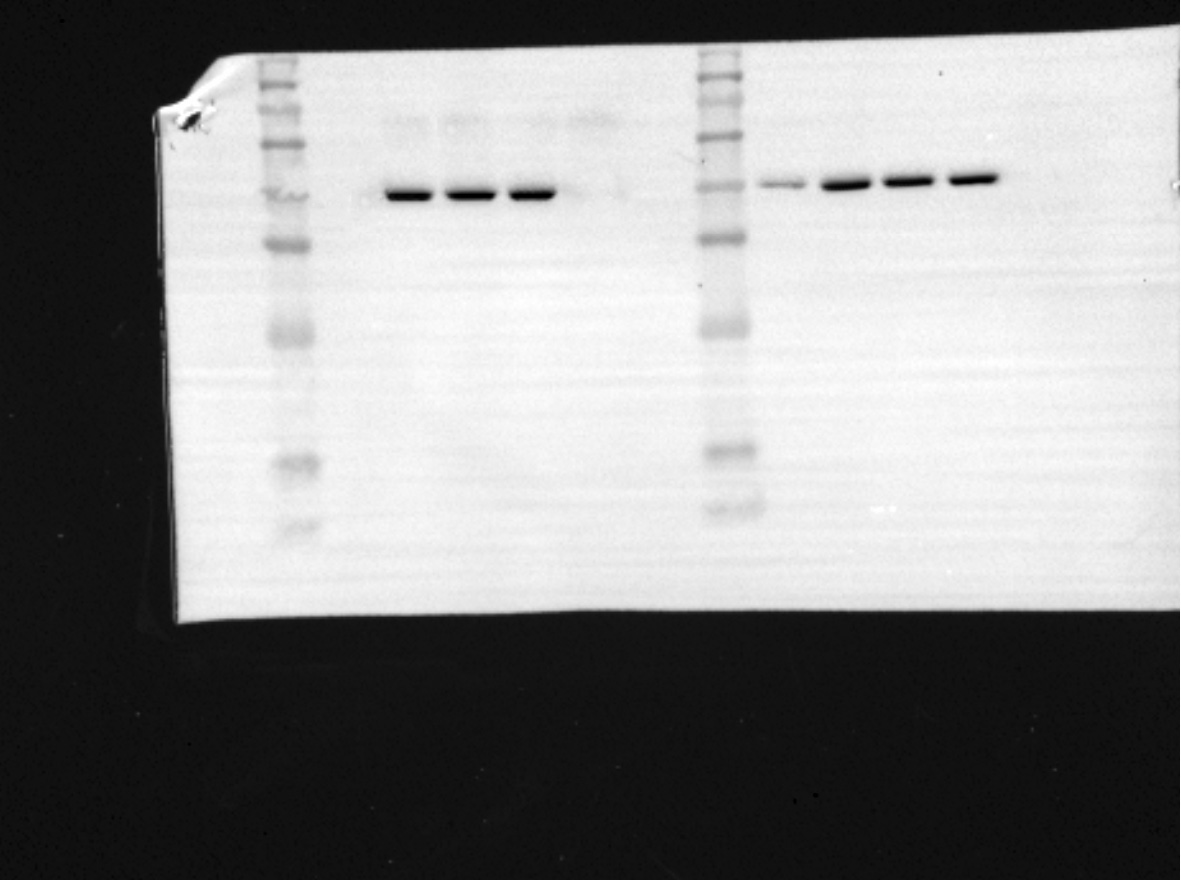

Supplement: Supplementary file 1 [file DataSheet_1.zip › raw data/WB/figure 3/A/YAP-actin.jpg]

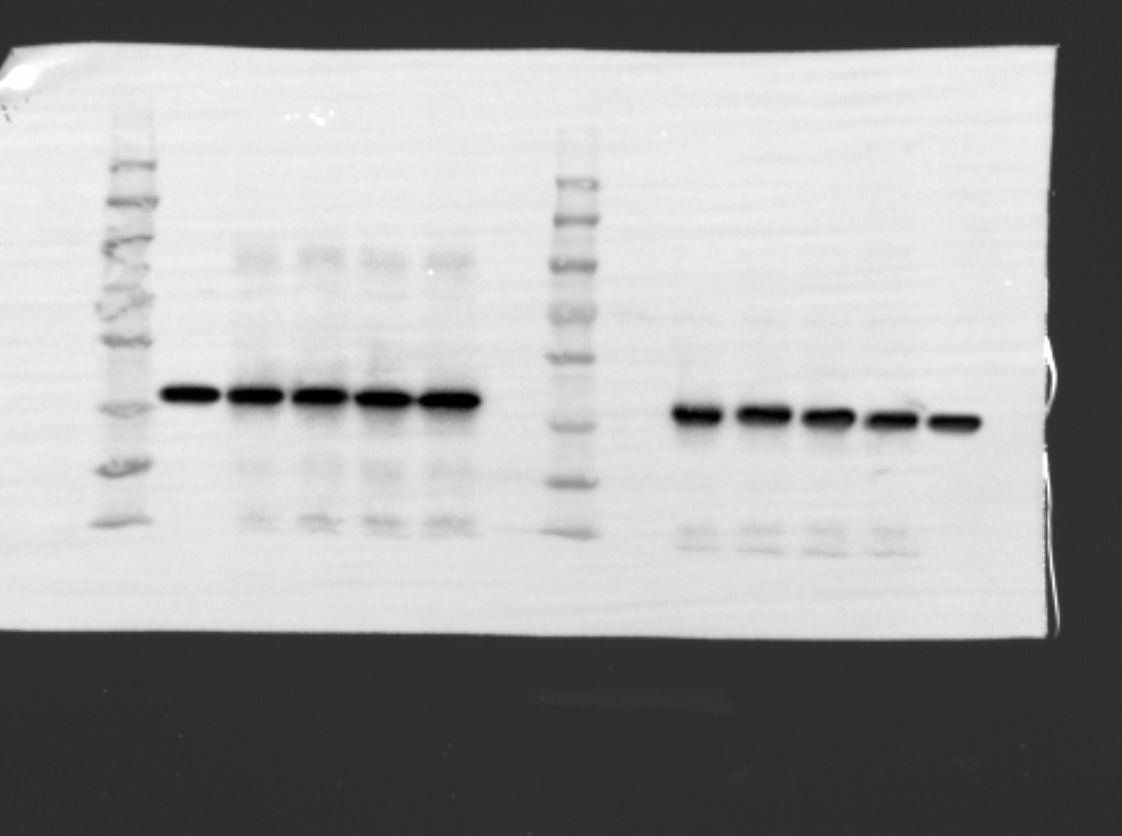

Supplement: Supplementary file 1 [file DataSheet_1.zip › raw data/WB/figure 3/B/A549-H1299-ACTIN.jpg]

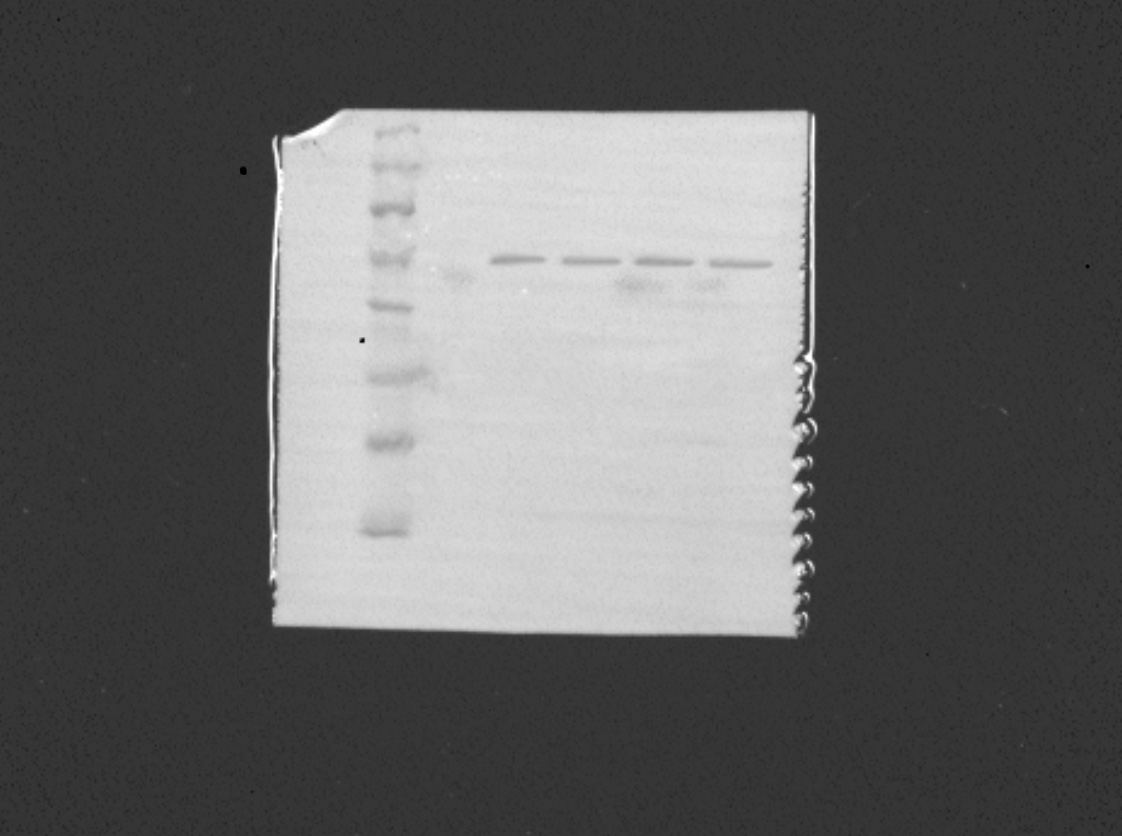

Supplement: Supplementary file 1 [file DataSheet_1.zip › raw data/WB/figure 3/B/A549KO-p-YAP.jpg]

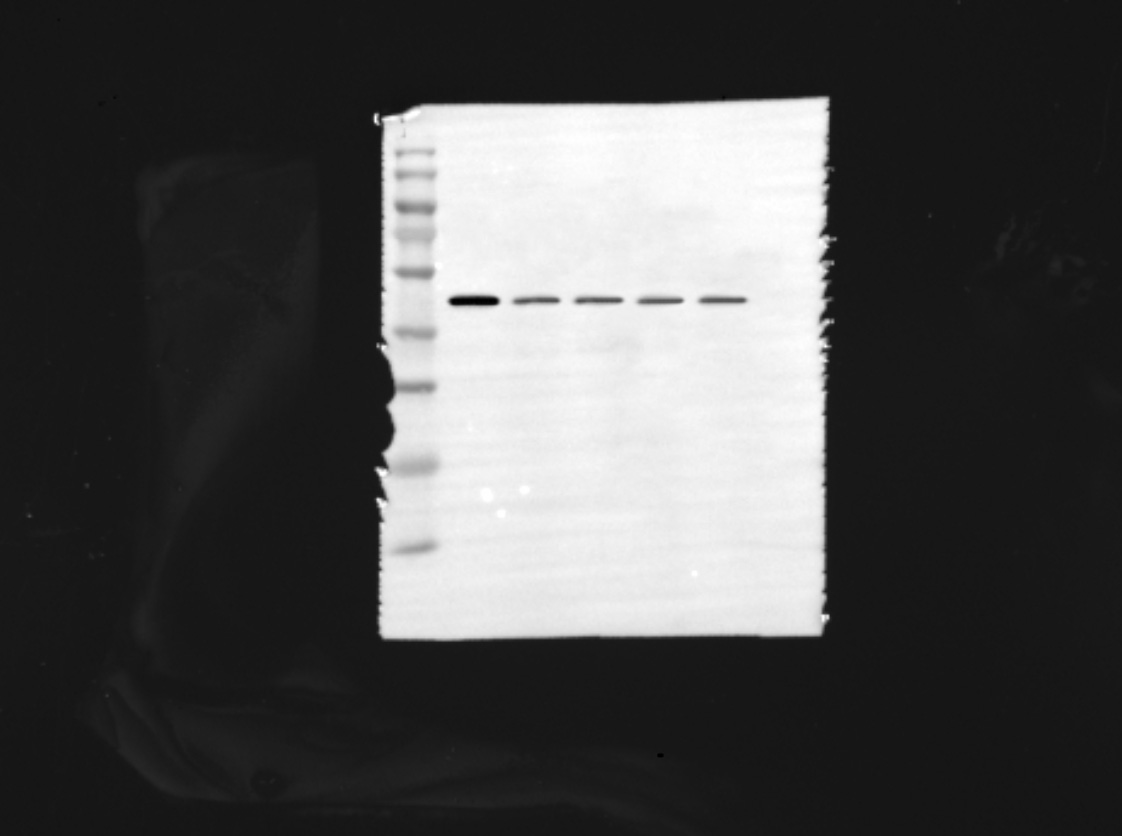

Supplement: Supplementary file 1 [file DataSheet_1.zip › raw data/WB/figure 3/B/A549KO-TAZ.jpg]

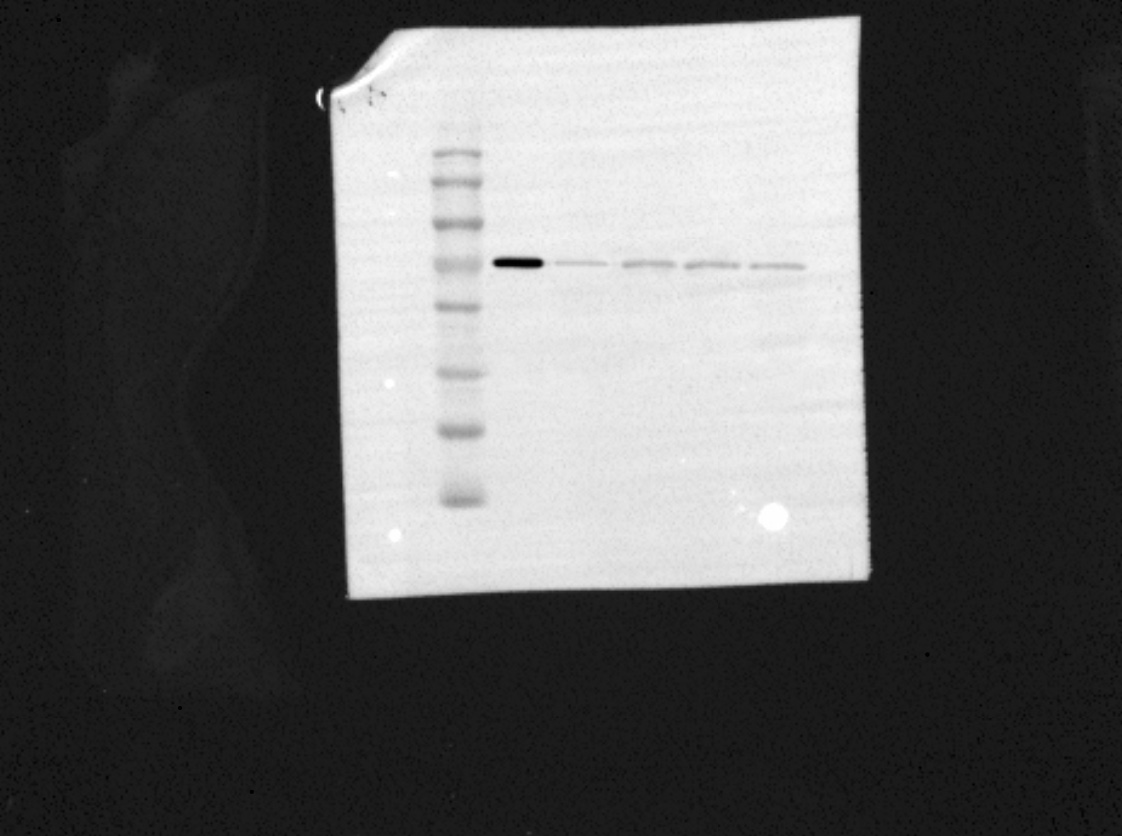

Supplement: Supplementary file 1 [file DataSheet_1.zip › raw data/WB/figure 3/B/A549-KO-YAP.jpg]

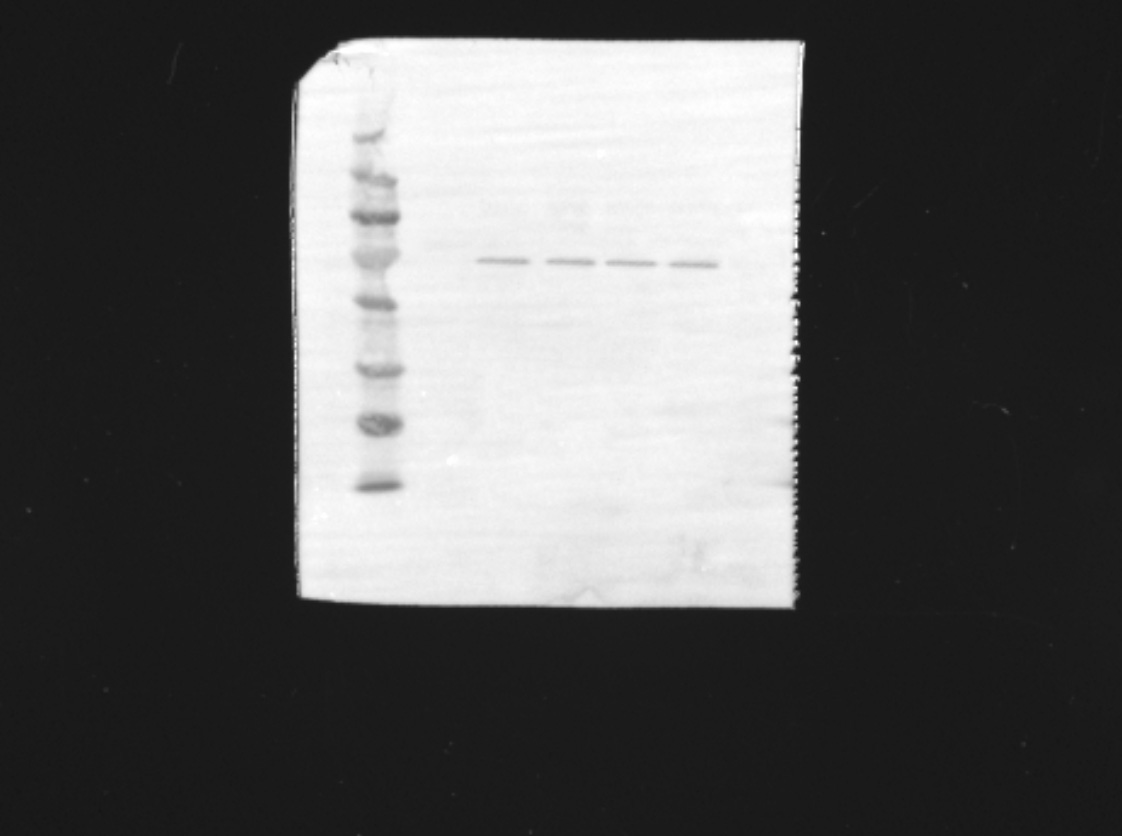

Supplement: Supplementary file 1 [file DataSheet_1.zip › raw data/WB/figure 3/B/h1299 ko-p-YAP.jpg]

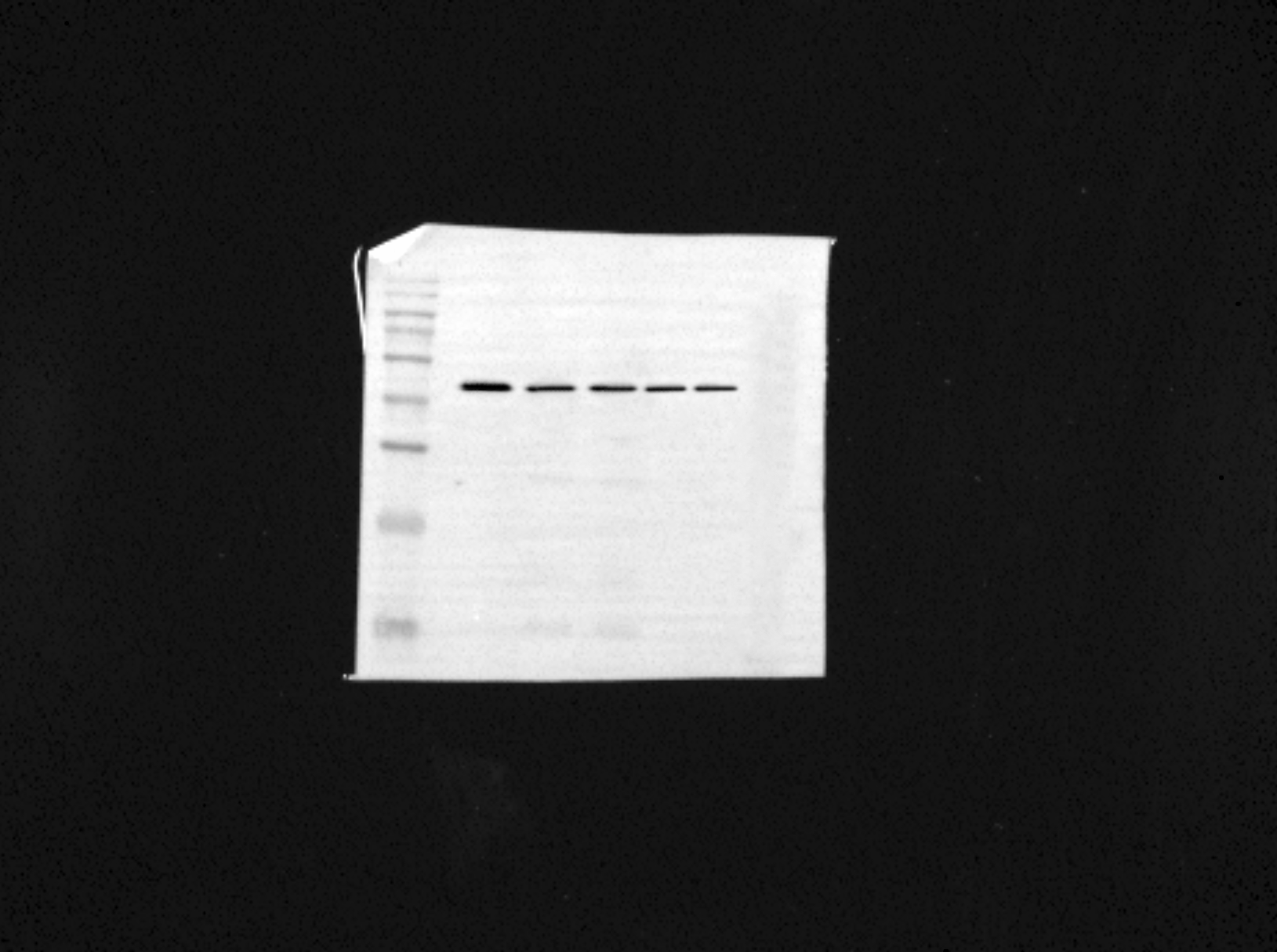

Supplement: Supplementary file 1 [file DataSheet_1.zip › raw data/WB/figure 3/B/H1299KO-TAZ.tif]

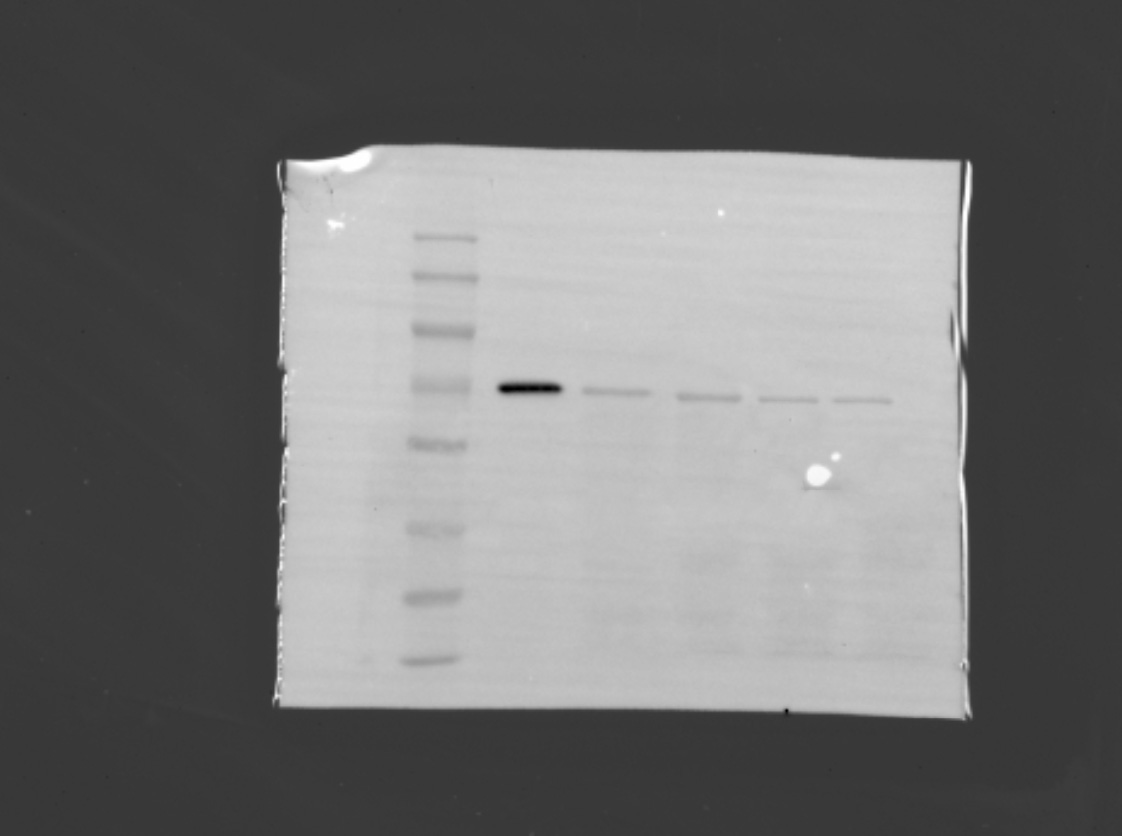

Supplement: Supplementary file 1 [file DataSheet_1.zip › raw data/WB/figure 3/B/h1299ko-YAP.jpg]

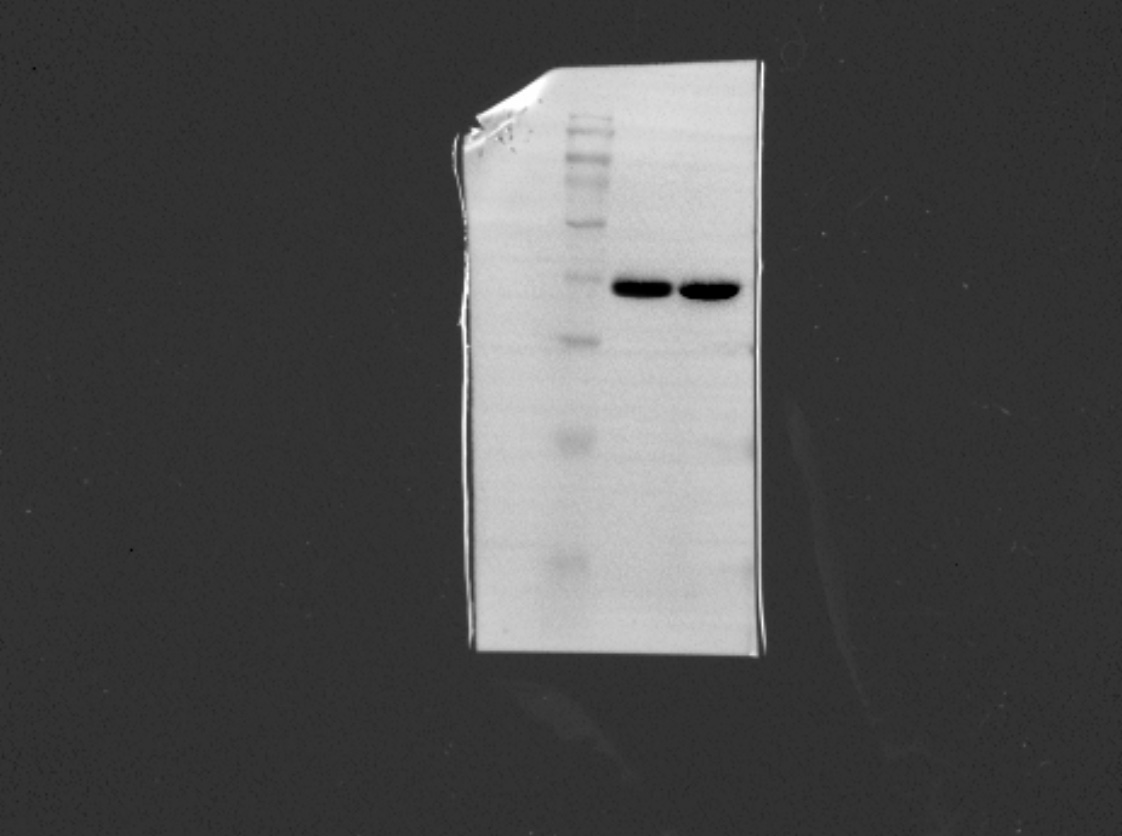

Supplement: Supplementary file 1 [file DataSheet_1.zip › raw data/WB/figure 3/F/patients actin.jpg]

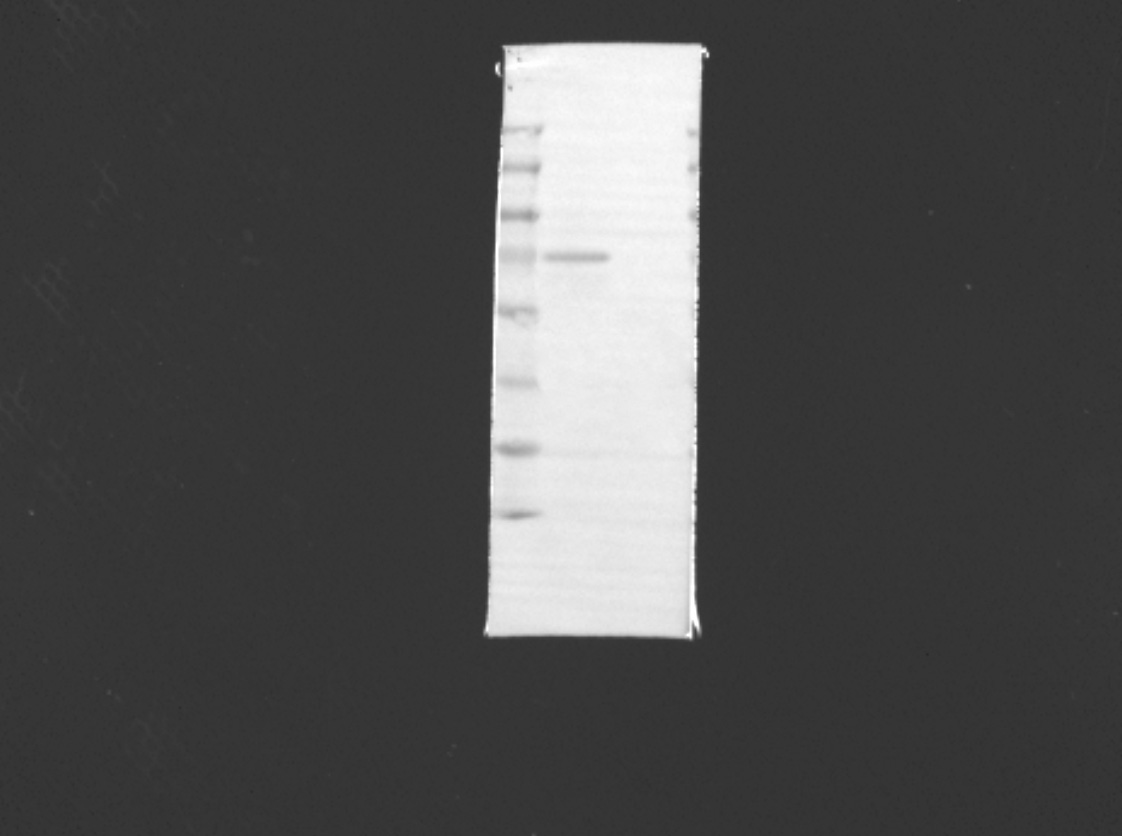

Supplement: Supplementary file 1 [file DataSheet_1.zip › raw data/WB/figure 3/F/patients-p-YAP.jpg]

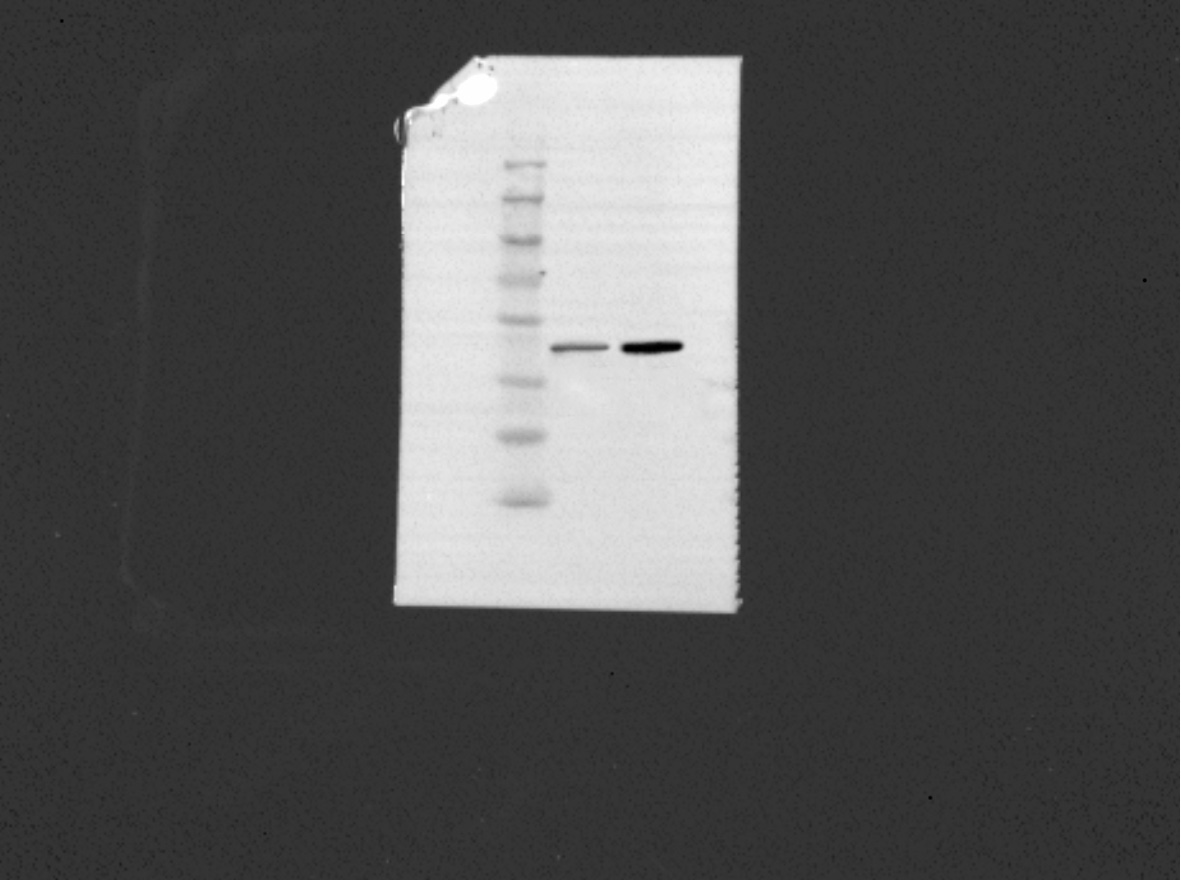

Supplement: Supplementary file 1 [file DataSheet_1.zip › raw data/WB/figure 3/F/patients-TAZ.jpg]

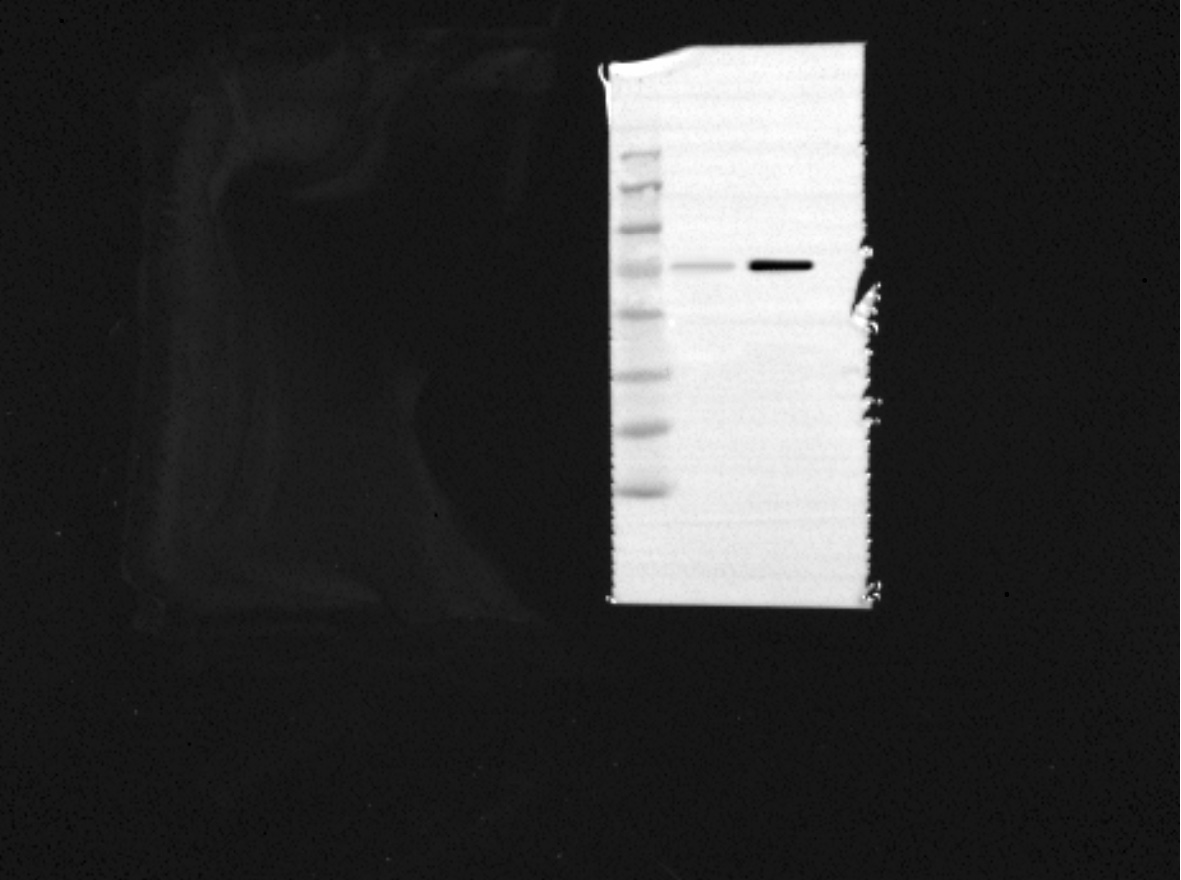

Supplement: Supplementary file 1 [file DataSheet_1.zip › raw data/WB/figure 3/F/patients-YAP.jpg]

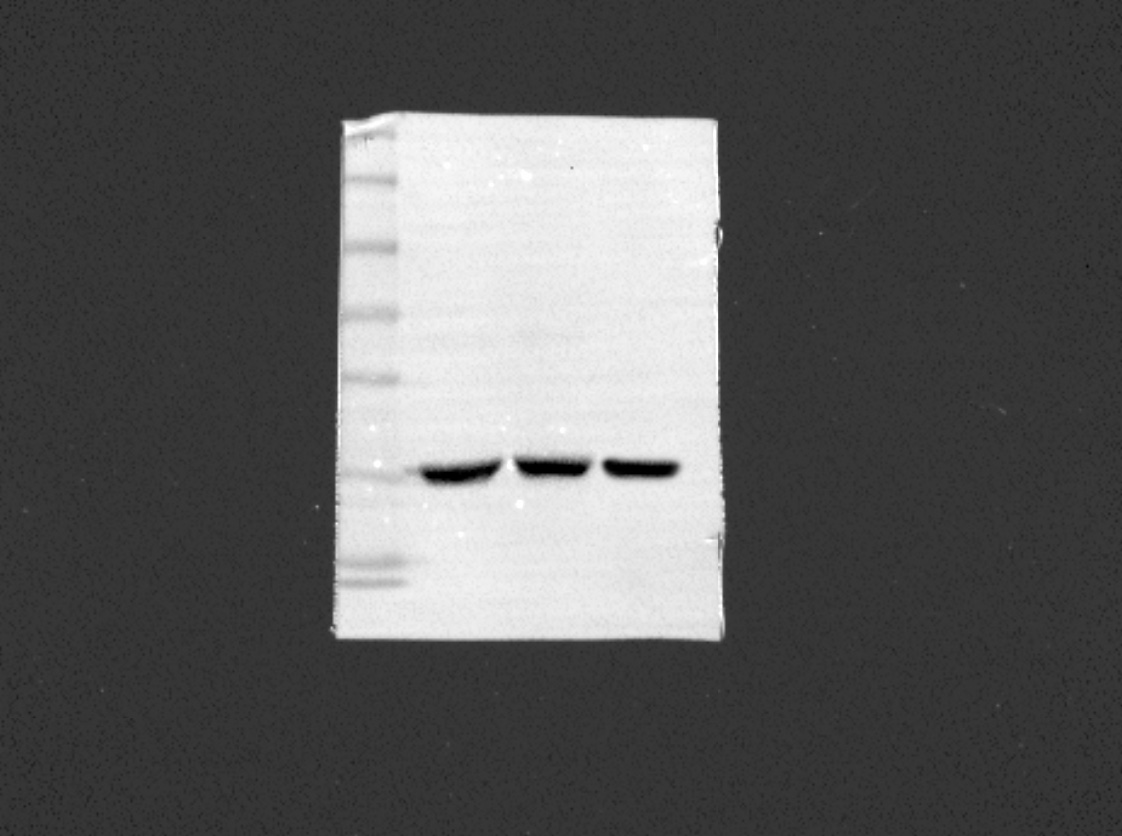

Supplement: Supplementary file 1 [file DataSheet_1.zip › raw data/WB/figure 4/A/A549-ACTIN.jpg]

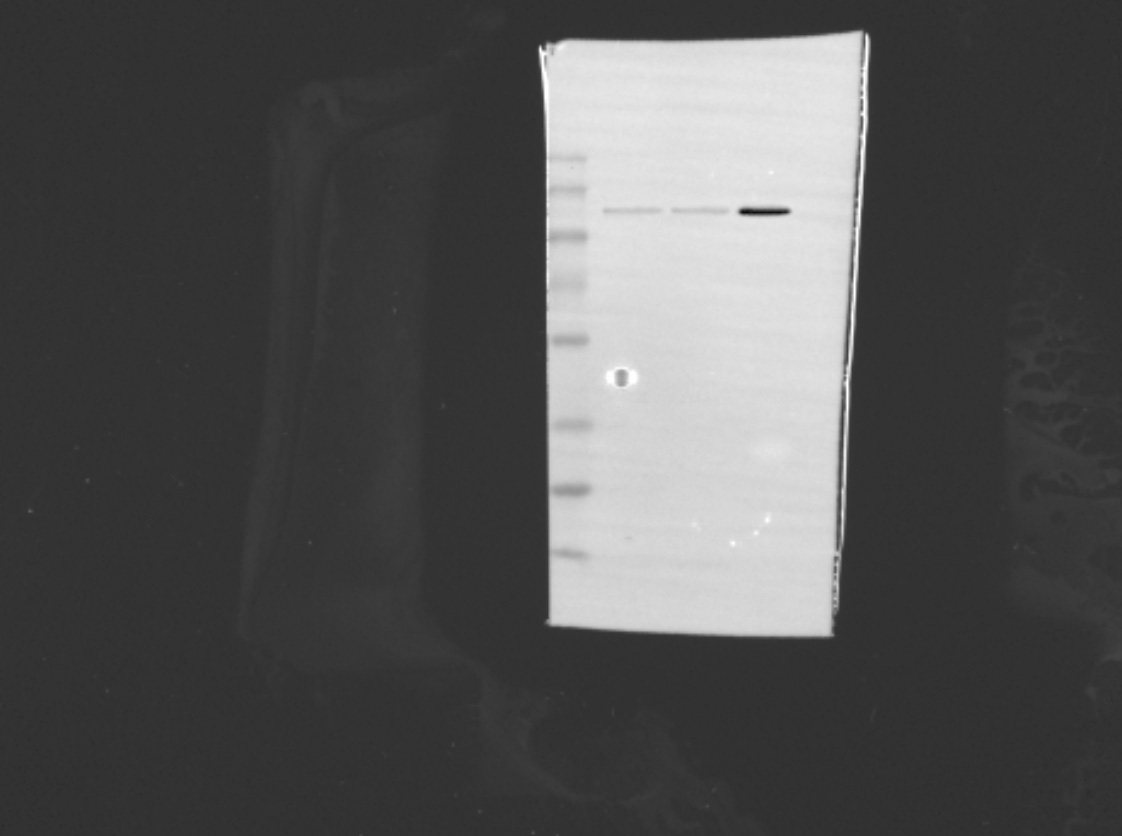

Supplement: Supplementary file 1 [file DataSheet_1.zip › raw data/WB/figure 4/A/A549-p-FAK.jpg]

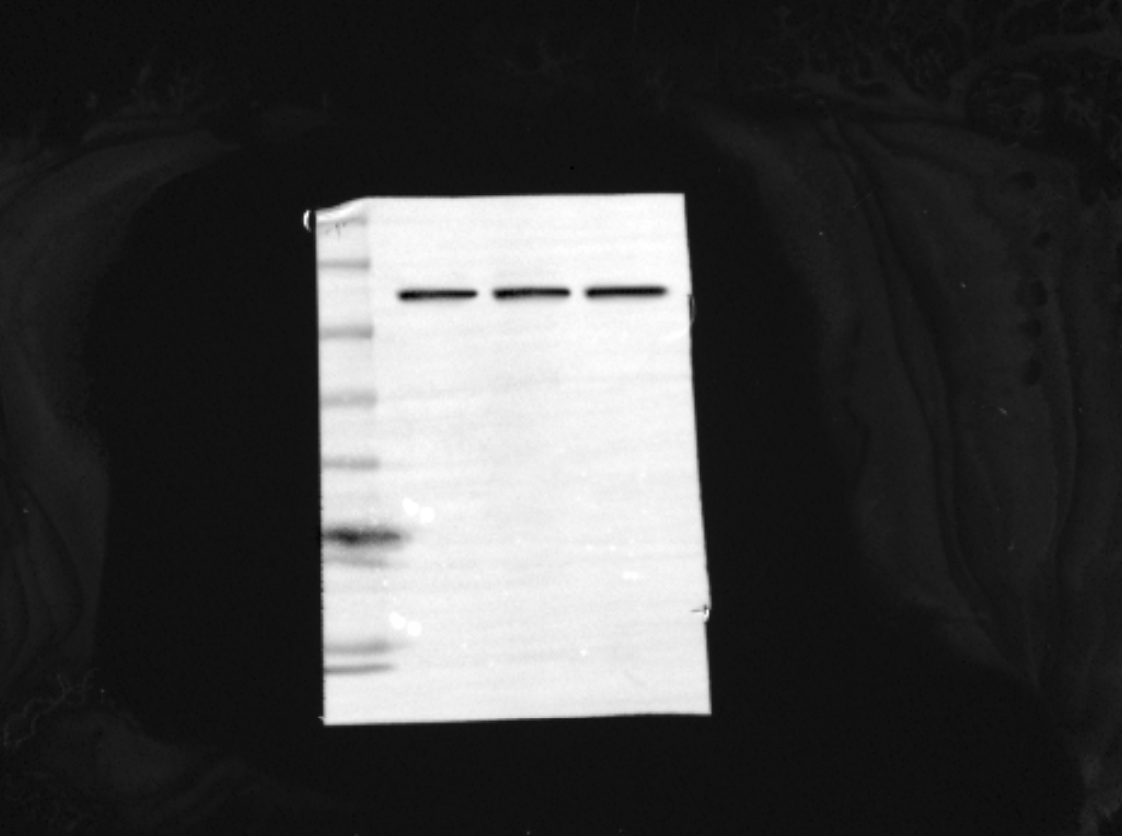

Supplement: Supplementary file 1 [file DataSheet_1.zip › raw data/WB/figure 4/A/A549-t-FAK.tif]

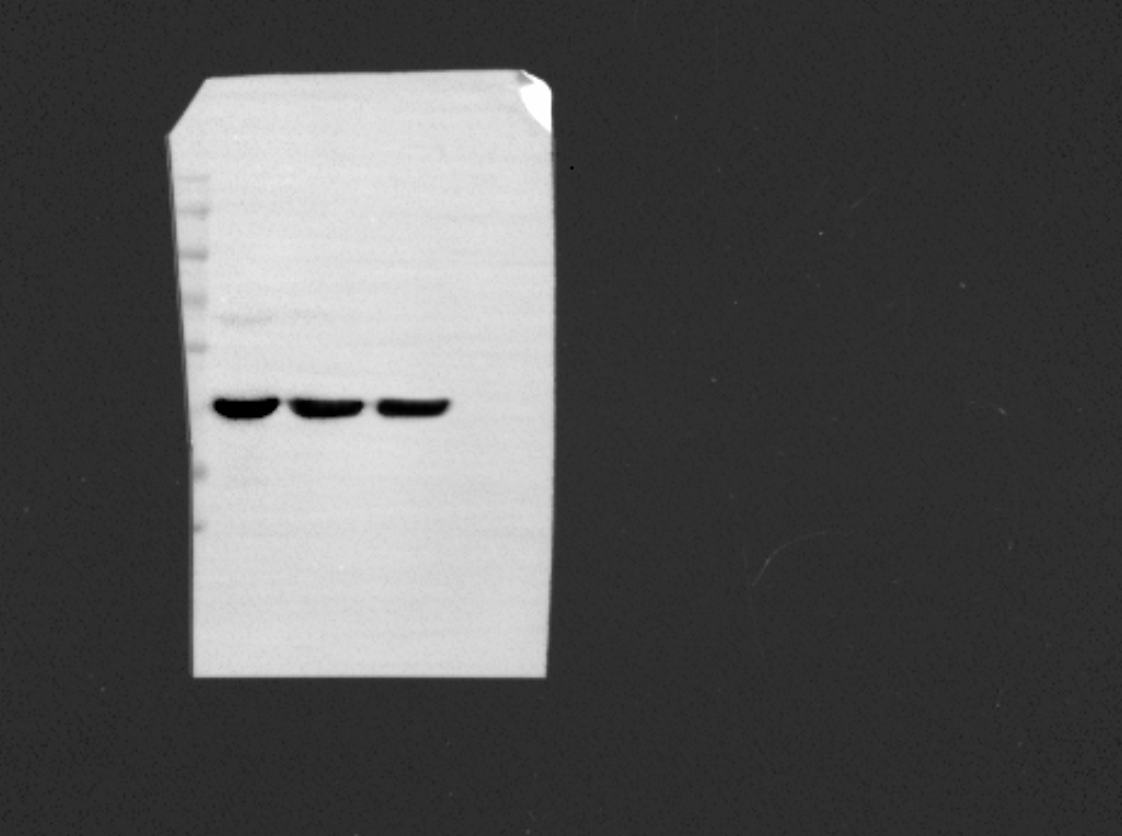

Supplement: Supplementary file 1 [file DataSheet_1.zip › raw data/WB/figure 4/A/H1299 actin.tif]

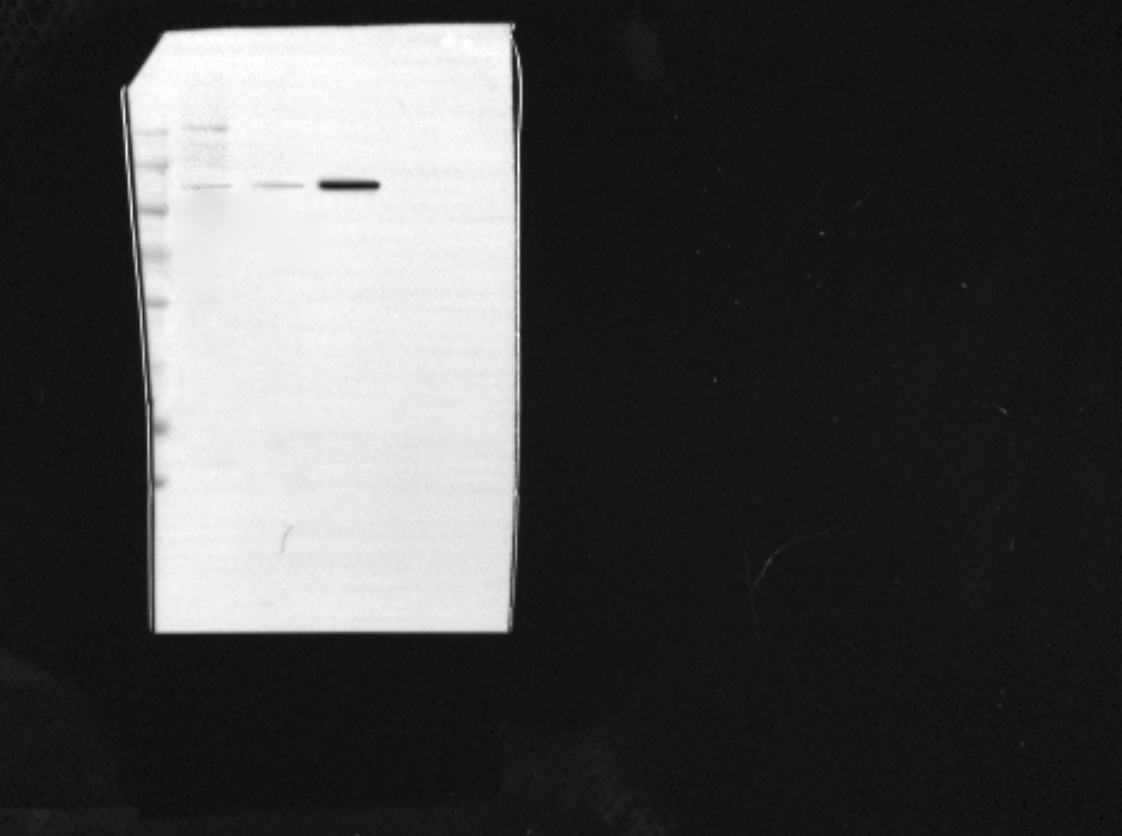

Supplement: Supplementary file 1 [file DataSheet_1.zip › raw data/WB/figure 4/A/h1299-p-FAK.tif]

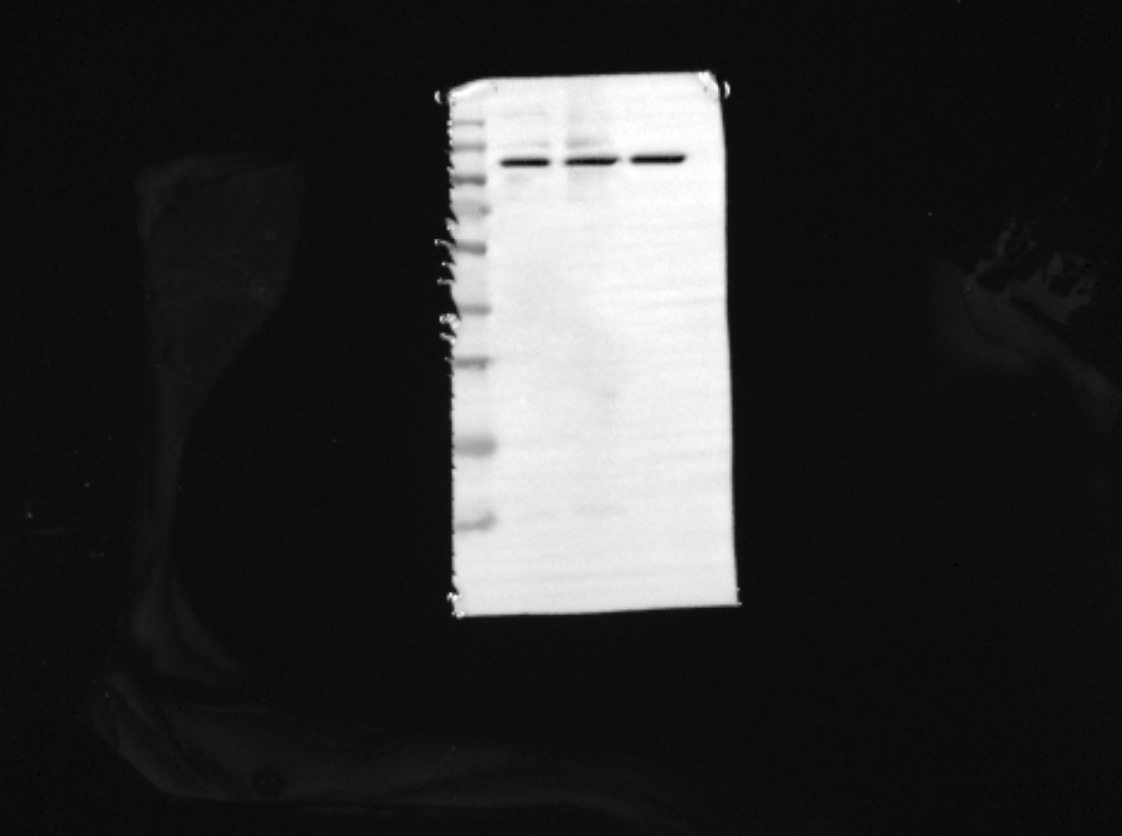

Supplement: Supplementary file 1 [file DataSheet_1.zip › raw data/WB/figure 4/A/H1299-t-FAK.jpg]

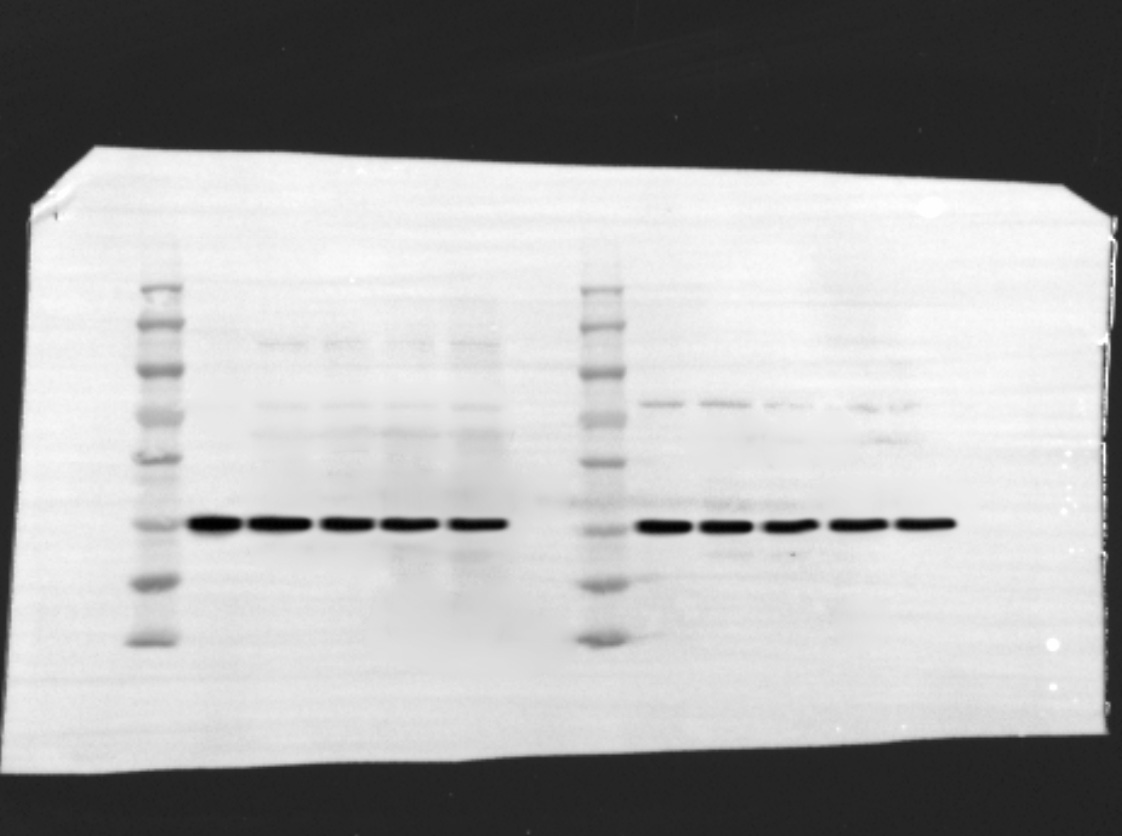

Supplement: Supplementary file 1 [file DataSheet_1.zip › raw data/WB/figure 4/B/A549-H1299K0-actin.jpg]

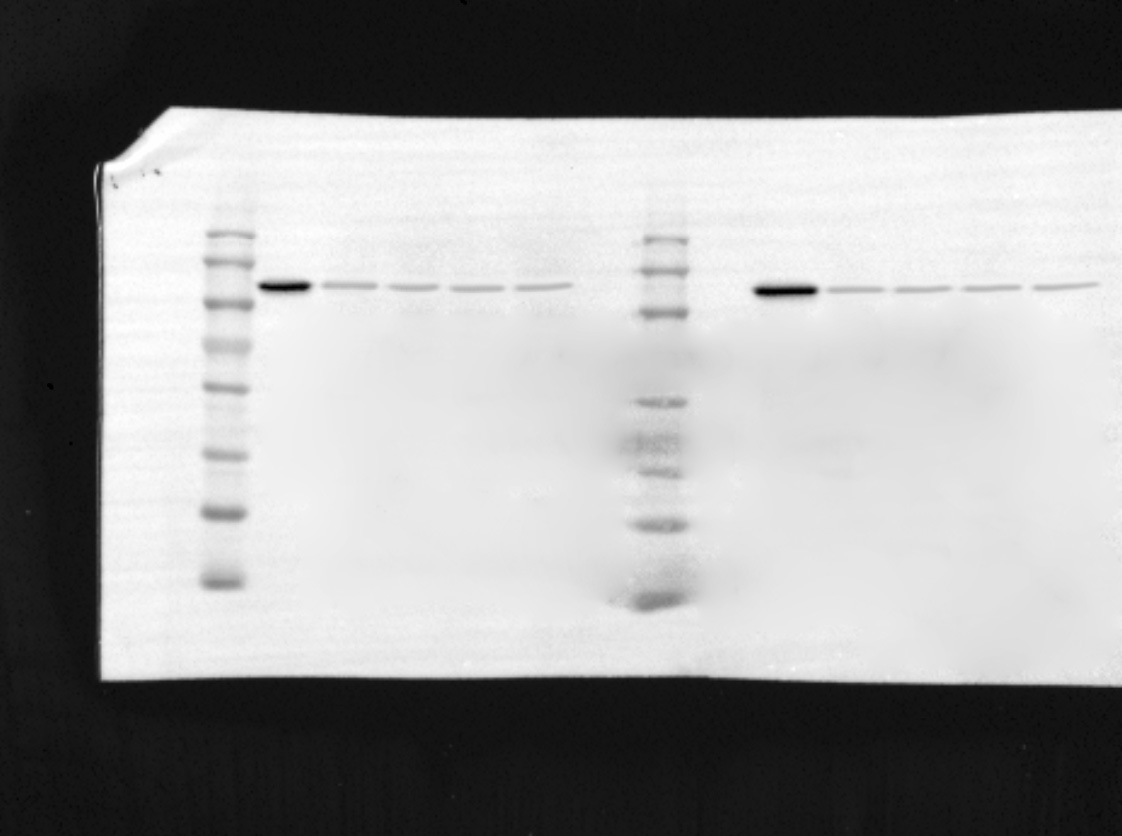

Supplement: Supplementary file 1 [file DataSheet_1.zip › raw data/WB/figure 4/B/A549KO-p-FAK.jpg]

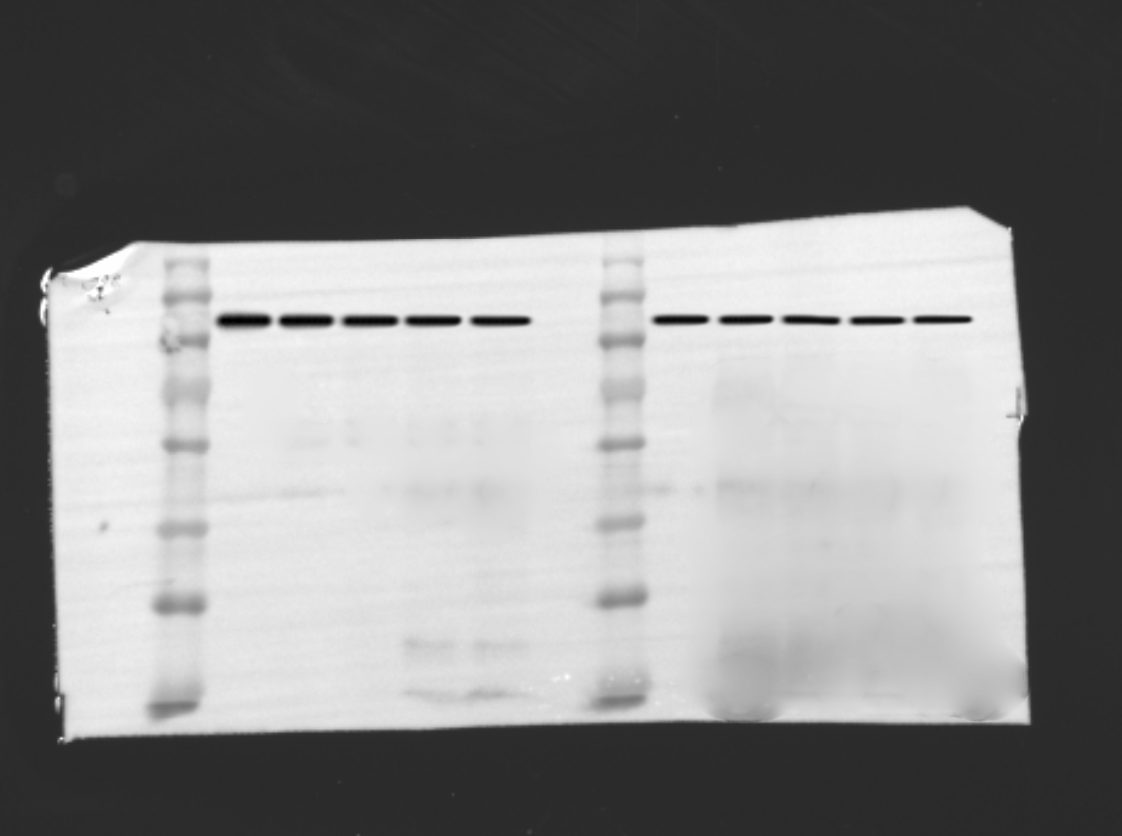

Supplement: Supplementary file 1 [file DataSheet_1.zip › raw data/WB/figure 4/B/t-FAK.tif]

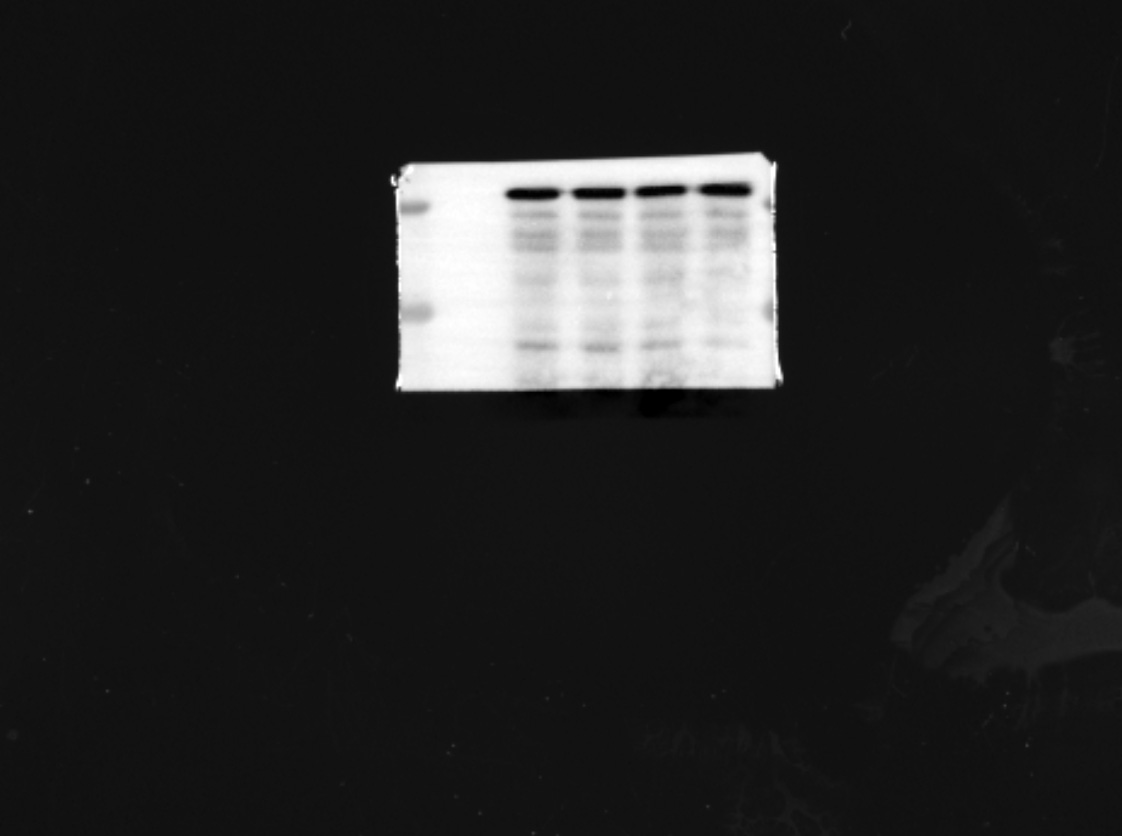

Supplement: Supplementary file 1 [file DataSheet_1.zip › raw data/WB/figure 4/C/actin.jpg]

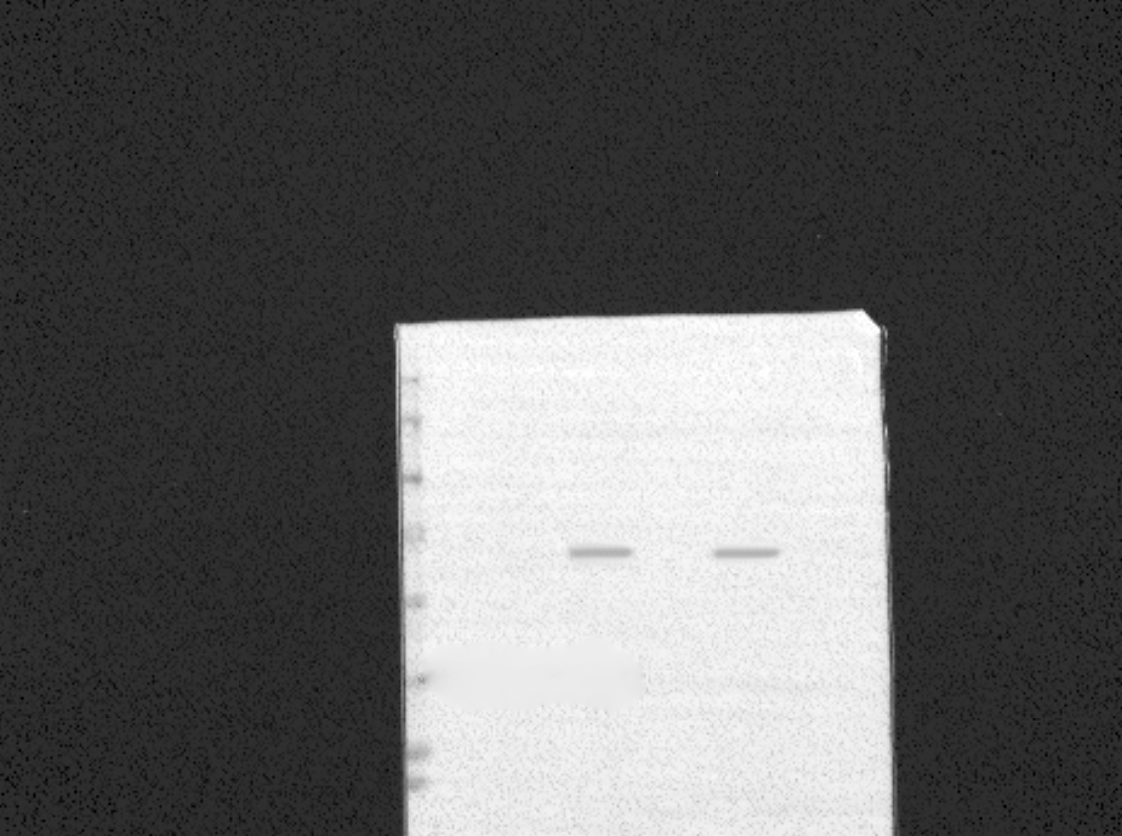

Supplement: Supplementary file 1 [file DataSheet_1.zip › raw data/WB/figure 4/C/p-YAP.tif]

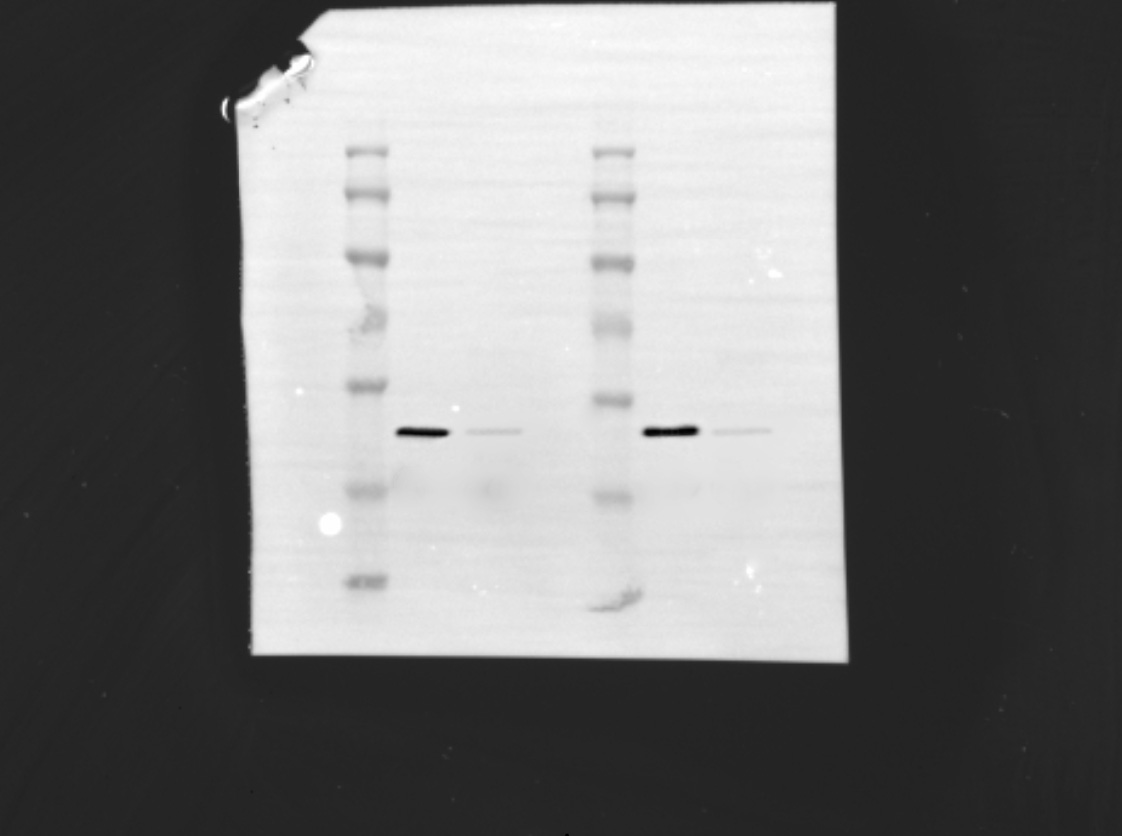

Supplement: Supplementary file 1 [file DataSheet_1.zip › raw data/WB/figure 4/C/TAZ.jpg]

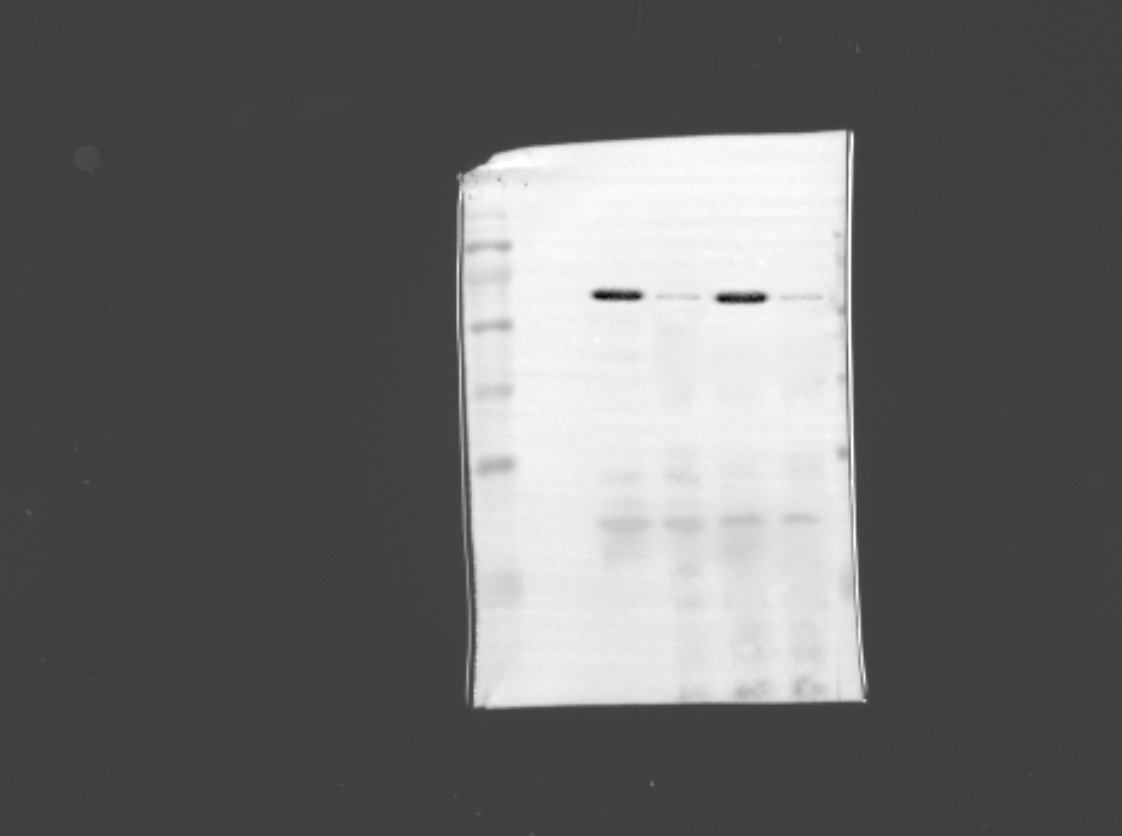

Supplement: Supplementary file 1 [file DataSheet_1.zip › raw data/WB/figure 4/C/YAP.jpg]
