# Supplementary material for: Laminin-bound integrin α6β4 promotes non-small cell lung cancer progression via the activation of YAP/TAZ signaling pathway
Source: Front Oncol. 2022 Oct 6;12:1015709. doi: 10.3389/fonc.2022.1015709 (PMC9583390; doi:10.3389/fonc.2022.1015709)
Supplement: Supplementary file 2 [file Table_1.docx]

Supplementary table 1 Primer sequence and Tm of each gene.

| Gene name | Primer sequence (5’-3’) | Tm |
| --- | --- | --- |
| human integrin β1 | 5′-CCTACTTCTGCACGATGTGATG-3′  5′-CCTTTGCTACGGTTGGTTACATT-3′ | 60℃ |
| human integrin β4 | 5′-GCAGCTTCCAAATCACAGAGG-3′  5′-CCAGATCATCGGACATGGAGTT-3′ | 60℃ |
| human integrin α3 | 5′-TCAACCTGGATACCCGATTCC-3′  5′-GCTCTGTCTGCCGATGGAG-3′ | 60℃ |
| human integrin α5 | 5′-GGCTTCAACTTAGACGCGGAG-3′  5′-TGGCTGGTATTAGCCTTGGGT-3′ | 61℃ |
| human integrin α6 | 5′-ATGCACGCGGATCGAGTTT-3′  5′-TTCCTGCTTCGTATTAACATGCT-3′ | 60℃ |
| human GAPDH | 5'-GGAGCGAGATCCCTCCAAAAT-3'  5'-GGCTGTTGTCATACTTCTCATGG-3' | 60℃ |
